# Development of a novel testis-on-a-chip that demonstrates reciprocal crosstalk between Sertoli and Leydig cells in testicular tissue

**DOI:** 10.1038/s12276-024-01258-3

**Published:** 2024-07-01

**Authors:** Se-Ra Park, Myung Geun Kook, Soo-Rim Kim, Choon-Mi Lee, Jin Woo Lee, Jung-Kyu Park, Chan Hum Park, Byung-Chul Oh, YunJae Jung, In-Sun Hong

**Affiliations:** 1 Department of Health Sciences and Technology, GAIHST, Gachon University, Incheon, 21999 Republic of Korea; 2 Department of Molecular Medicine, School of Medicine, Gachon University, Incheon, 406-840 Republic of Korea; 3 Department of Otolaryngology-Head and Neck Surgery, Chuncheon Sacred Heart Hospital, Hallym University College of Medicine, Chuncheon, Republic of Korea; 4 Department of Physiology, Lee Gil Ya Cancer and Diabetes Institute, Gachon University College of Medicine, Incheon, 21999 Republic of Korea; 5 Department of Microbiology, College of Medicine, Gachon University, Incheon, 21999 Korea

**Keywords:** Biomarkers, Endocrine system and metabolic diseases

## Abstract

The reciprocal crosstalk between testicular Sertoli and Leydig cells plays a vital role in supporting germ cell development and maintaining testicular characteristics and spermatogenesis. Conventional 2D and the recent 3D assay systems fail to accurately replicate the dynamic interactions between these essential endocrine cells. Furthermore, most in vitro testicular tissue models lack the ability to capture the complex multicellular nature of the testis. To address these limitations, we developed a 3D multicellular testis-on-a-chip platform that effectively demonstrates the reciprocal crosstalk between Sertoli cells and the adjacent Leydig cells while incorporating various human testicular tissue constituent cells and various natural polymers infused with blood coagulation factors. Additionally, we identified SERPINB2 as a biomarker of male reproductive toxicity that is activated in both Sertoli and Leydig cells upon exposure to various toxicants. Leveraging this finding, we designed a fluorescent reporter-conjugated toxic biomarker detection system that enables both an intuitive and quantitative assessment of material toxicity by measuring the converted fluorescence intensity. By integrating this fluorescent reporter system into the Sertoli and Leydig cells within our 3D multicellular chip platform, we successfully developed a testis-on-chip model that can be utilized to evaluate the male reproductive toxicity of potential drug candidates. This innovative approach holds promise for advancing toxicity screening and reproductive research.

## Innovative ‘testis-on-chip’ model advances drug candidate toxicity screening

Spermatogenesis, or sperm creation, happens in the testis and involves various cells, including Sertoli and Leydig cells. However, traditional single-cell-based 2D assay models (tests that measure the presence of a substance) don’t accurately show the complex interactions between these cells. To solve this, scientists created a ‘human testis-on-a-chip’ platform that imitates the complex cell interactions and hormone communication of seminiferous tubules (small tubes) in the testis. The chip was made using polydimethylsiloxane (a type of silicone) and included multiple testicular tissue cells. The scientists found that the chip could keep the cells alive and active for up to 28 days. Also, the chip was able to produce hormones and respond to hormonal stimulation. This study provides a useful tool for studying male reproductive biology and testing potential drugs. This summary was initially drafted using artificial intelligence, then revised and fact-checked by the author.

## Introduction

Spermatogenesis occurs in the seminiferous tubules, which harbor germ cells and Sertoli cells, within the testis^1^. Sertoli cells (a kind of “nurse” cell) play a key role in regulating spermatogenesis by forming the blood-testis barrier^2^ and secreting various nutrients, hormones, and growth factors^3^ into the seminiferous tubules. The adjacent Leydig cells support spermatogenesis as a primary source of testosterone or endocrine factors and are located in the connective tissue surrounding the seminiferous tubules^4^. Sertoli cells produce various factors that regulate steroidogenesis in Leydig cells and the functions of these steroids^5,6^. Therefore, the functional interactions between these two types of cells play essential roles in regulating spermatogenesis, and dysfunction in their crosstalk may cause several male reproductive abnormalities, such as cryptorchidism^7^, hypospadias^8^, and hypogonadism^9,10^. On the other hand, traditional single-cell-based 2D assay models do not properly reflect the multicellular complexity or reciprocal intercellular communication in human seminiferous tubules. In this context, many studies have developed complex 3D structures using testicular cells to mimic the in vivo microenvironment of seminiferous tubules. Nevertheless, the vast majority of these studies are based on animal cells^11–15^, and the few studies using human testicular cells^16^ were unable to properly reflect the functional interactions between Sertoli and Leydig cells or with other testicular constituent cells.

Therefore, the human testis-on-a-chip was developed as a novel in vitro assay system that can properly epitomize multicellular complexity and reciprocal crosstalk between the seminiferous tubules of the testis by incorporating multiple testicular tissue constituent cells and 3D structures to incorporate a natural polymer mixture into the testis-on-a-chip platform. In more detail, the chip platform consisted of dual interacting two tissue compartments of seminiferous tubules: the Sertoli chamber and the Leydig chamber. Human Sertoli and Leydig cells were loaded into the corresponding chambers of the testis-on-a-chip platform with a natural polymer mixture (collagen and hyaluronic acid). In addition, the Sertoli and Leydig chambers were interconnected by vessel cell-coated media channels, which allowed reciprocal endocrine crosstalk between them via the exchange of various growth factors, steroid hormones, and endocrine factors. Furthermore, the outer space of the two chambers was loaded with vascular endothelial cells and immune cells (macrophages) with the aforementioned natural polymer mixture to reflect the complex microenvironment of seminiferous tubules that interact with various endocrine or nonendocrine cell types.

Although these natural polymer-based tissue architectures within the chip platform have high biocompatibility, their mechanical strength is insufficient to exert the necessary therapeutic effects because they lack the ability to form covalent crosslinks^17^. Thus, various chemical crosslinking agents have been incorporated to improve the mechanical strength and structural stability of natural polymer-based tissue architectures^18^. However, a significant disadvantage of chemical-based polymerization strategies is the potential cytotoxicity of the residual unreacted crosslinking agents^19^, which limits their clinical application. Therefore, nontoxic blood coagulation factors (fibrinogen and thrombin) were used to increase the mechanical strength of the tissue architecture to make natural polymers within the chip platform without residual toxicity.

Early changes in signaling activity or gene expression caused by exposure to toxins are more likely to be associated with the onset of toxicity than higher threshold final stage functional events, such as apoptotic cell death and growth arrest. Hence, measuring these early changes is a more sensitive way in which prompt information on the toxicity of drug candidates or specific materials can be obtained^20^. Therefore, early signaling pathways or genes involved in the response to toxin exposure can serve as biomarkers of toxicity. A toxicity biomarker-based screening platform was developed by RNA sequencing in Sertoli and Leydig cells with or without exposure to toxins. SERPINB2 (also known as plasminogen activator inhibitor type 2) can be a reliable biomarker that can predict male reproductive toxicity, such as apoptotic cell death and growth arrest, in both cell types. A fluorescent gene (GFP or mCherry) was incorporated into the prompter region of the identified toxicity biomarker SERPINB2 to intuitively and quantitatively evaluate the male reproductive toxicity of specific materials. The fluorescent gene-incorporated reporter system was then introduced into the loaded Sertoli and Leydig cells within each chamber of the chip platform. This system made detecting the male reproductive toxicity of specific materials possible by measuring the fluorescence intensity. To the best of the authors’ knowledge, this is the first study to develop a testis-on-a-chip using reciprocally communicating human Sertoli and Leydig cells with a fluorescent reporter-conjugated toxicity biomarker-based detection system. This testis-on-a-chip can also provide valuable information on newly developed drug candidates whose toxicities are not detected by traditional single-cell monolayer-based or even recent 3D-based screening platforms.

## Materials and methods

### Isolation and establishment of various human testicular cellular components

Normal testicular tissue was obtained from a patient who underwent bilateral orchiectomy with complete spermatogenesis at the Gachon University Gil Medical Center; written informed consent was obtained from the patient, and the study was approved by the Gachon University Institutional Review Board (IRB no: 1044396-202106-HR-118-01). The testicular tissue was minced mechanically into small pieces. The small pieces were then digested enzymatically in DMEM containing 10% fetal bovine serum (FBS) and 250 U/ml type I collagenase for 5 h at 37 °C in a rotating shaker. The digested solution was filtered through a cell strainer (mesh size 70 µm) to remove the remaining undigested tissue fragments. The filtrate was then filtered through a 40 µm mesh cell strainer to separate the Sertoli and Leydig cell populations from the vascular cells and their aggregates. The isolated Sertoli and Leydig cells were cultured in a ScienCell (cat. no.: 4521) with a Sertoli Cell Growth Supplement (ScienCell, cat. no.: 4572) and 10% FBS at 37 °C under 5% CO_2_.

### The testis-on-a-chip platform was fabricated by injecting polydimethylsiloane (PDMS) into a 3D-printed casting mold

**Fig. 1:**
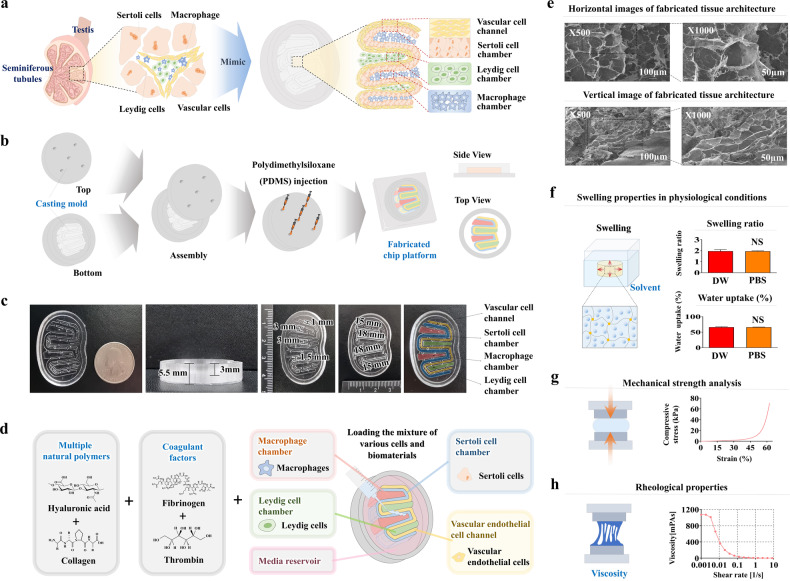
Development of human testis-on-a-chip that demonstrates reciprocal endocrine crosstalk between Sertoli and Leydig cells in the testicular tissue and analysis of its physical properties. The male seminiferous tubule is composed of two major endocrine cells, Sertoli and Leydig cells, which are reciprocally controlled by reproductive steroids and various endocrine factors. The Sertoli and Leydig cell chambers within the testis-on-a-chip were interconnected by vessel cell-coated media channels to allow reciprocal endocrine crosstalk. In addition, an immune cell (macrophage) chamber was incorporated into the chip platform to mimic the immune cell-interacting microenvironment of the seminiferous tubules (**a**). The casting mold for the human testis-on-a-chip platform that could properly mimic the structural features of the seminiferous tubules was fabricated using PLA-based 3D printing. Polydimethylsiloxane (PDMS) was then injected into the fabricated casting mold, and the synthesized chip platform was released from the mold after polymerization (**b**). The testis-on-a-chip platform was oval-shaped with a major axis length of 45 mm, center diameter of 30 mm, and height of 5.5 mm and was fabricated to mimic the microenvironment of the seminiferous tubules and endocrine crosstalk between the Sertoli and Leydig cell chambers (**c**). To reflect the multicellular complexity and physiological features of testicular tissue, various human testicular cellular components (Sertoli cells, Leydig cells, macrophages, and vascular endothelial cells) with a natural polymer mixture (collagen and hyaluronic acid) and blood coagulation factors (fibrinogen and thrombin) were loaded into the fabricated testis-on-a-chip platform. Two major human testicular cell types, Sertoli and Leydig cells, were isolated from a patient who underwent bilateral orchiectomy with complete spermatogenesis (**d**). The horizontal and vertical scanning electron microscopy (SEM) images of the fabricated natural polymer-based tissue architecture revealed a uniformly interconnecting microporous structure with different pore diameters ranging from 50 to 150 μm (**e**). The swelling behavior of the fabricated tissue architecture was evaluated by imaging all the samples in DW and PBS (pH 7.4) in each well at 37 °C. Nonabsorbed water was removed from the samples, and the hydrated samples were weighed to determine their water absorption potential (**f**). The mechanical strength of the fabricated natural polymer-based tissue architecture was measured by applying uniaxial compressive stress with a tensile strength testing machine (QM100S, QMESYS, Gunpo, Korea). The fabricated tissue architectures (10 mm in diameter and 10 mm in height) were analyzed to determine their mechanical strength. To determine the compressive stress at which the fracture occurred, compressive stress was applied at a loading rate of 5 mm/min until the samples were fractured (**g**). The rheological properties (viscosity) of the tissue architectures were analyzed by varying the shear rate from 1 s^−1^ to 10 s^−1^, which allows assessment of the shear thinning or thickening behavior (**h**). Significant differences are indicated as follows: *p < 0.05, **p < 0.005, and ***p < 0.001 (two-sample *t* test).

As shown in Fig. 1b, computer-aided design (CAD) software was used to design the casting mold for the testis-on-a-chip. After finishing the design, the 3D CAD data were converted to 3D surface geometric data composed of many different types of vectors and then converted to the separated data at the Z-axis with a thickness of 50 μm. The information from each separated layer was transferred to a digital light processing (DLP)-based 3D printer (Master EV, Carima, Seoul, Korea), and the casting mold for testis-on-a-chip was manufactured from poly(lactic) acid (PLA) using a layer-by-layer process (Fig. 1b).

### Manufacturing process for the PDMS-based testis-on-a-chip platform

PDMS (Sylgard® 184, Dow Corning Corp., Auburn, MI, USA) was prepared with of base and curing agent at a ratio of 20:3 with proper mixing to obtain homogenous polymerization after 10 minutes at room temperature. The PDMS mixed solution was injected into the 3D-printing casting mold for the testis-on-a-chip and placed in the vacuum chamber to remove the dissolved gases. Subsequently, the PDMS-injected casting mold was polymerized in an oven at 65 °C for 24 hours. After polymerization, the casting mold was cooled to room temperature. The fabricated chip was slowly released from the casting mold using a precision razor blade to avoid damaging the fabricated chip platform (Fig. 1d).

### Embedding various types of testicular tissue constituent cells in each compartment of the fabricated chip platform

Briefly, type I collagen (3 mg/ml) and hyaluronic acid (3 mg/ml) solutions (in DMEM) were prepared in specific growth media. The natural polymer mixture (composed of collagen and hyaluronic acid), fibrinogen (12.5 mg/ml), and thrombin (1.25 U/ml) solutions were also prepared in DMEM to improve the mechanical strength. Sertoli cell medium supplemented with Sertoli cell growth medium and 5% FBS plus 1% PS, which is widely used for culturing various human testicular cells, was selected as the common culture medium for human Sertoli and Leydig cells. Next, vascular endothelial cells, immune cells (macrophages), Sertoli cells, and Leydig cells were simultaneously reconstituted in a 1:1:1:1 mixture of hyaluronic acid (24 mg/ml), collagen (24 mg/ml), fibrinogen (50 mg/ml), and thrombin (5 U/ml) and then loaded into each compartment of the PDMS-based testis-on-a-chip platform without additional ECM coating using a syringe. For polymerization, the mixture was cooled at room temperature for 30 min. A Sertoli cell chamber (diameter: 1 mm, thickness: 3 mm) and a Leydig cell chamber (diameter: 1 mm, thickness: 3 mm) with cell densities of ~2 × 10^5^ cells/ml within the tissue construct mixed solution were obtained. To ensure uniformity in both the Sertoli and Leydig cell chambers, we maintained a consistent ratio of natural polymer complex, comprising type I collagen and hyaluronic acid, along with a mixture of fibrinogen and thrombin. Under static culture conditions, Sertoli cell medium supplemented with Sertoli cell growth supplement and 5% FBS plus 1% PS was added to the chip platform. The culture medium was changed every two to three days.

### Mechanical characterization of the fabricated testicular tissue architectures: morphology, compressive stress, and viscosity

Morphology: the microstructures of the natural polymer-based testicular tissue architectures were observed by SEM (VP-FE-SEM; EVO^®^LS10, Carl Zeiss, Germany) at the Korean Basic Science Institute (KBSI, Chuncheon, Korea). The fabricated testicular tissue architectures were freeze-dried, and the samples were coated with a thin (10 nm) layer of gold/palladium for 30 s at a discharge current of 15 mA (Ion Sputter 1010, Hitachi, Japan). Microstructural images were obtained at an accelerating voltage of 1.2–1.3 kV according to previously reported protocols^21^. Compressive stress: the compressive stress–strain curves of the testicular tissue architectures were obtained by applying uniaxial compression force with a universal testing machine (QM100S, QMESYS, Gunpo, Korea). Natural polymer-based testicular tissue architectures with dimensions of 10 mm (diameter) and 3 mm (height) were analyzed via compressive tests. A compression force was applied gradually at a displacement rate of 5 mm/min until the tissue architecture samples were fractured to calculate the stress at failure following previously reported protocols^21^. Viscosity: the rheological properties of the fabricated tissue architecture were analyzed at 37 °C on an Anton Paar MCR 102 rheometer (Anton Paar, Zofingen, Switzerland); the shear rate varied from 1 s^−1^ to 20 s^−1^ to allow an evaluation of the potential shear thinning or thickening behavior according to previously reported protocols^21^.

### Immunofluorescence staining

The samples were fixed with 4% paraformaldehyde for fluorescence staining. They were then permeabilized with 0.4 M glycine and 0.3% Triton X-100. Nonspecific binding was blocked with 2% normal swine serum (DAKO, Glostrup, Denmark). Staining was performed as described elsewhere^22^ using primary anti-mCherry (TAKARA and cat. no: 632524), anti-GFP (Invitrogen and Cat. No: V820-20), SOX9 (Abcam, cat. no.: ab185230), WT1 (Abcam, cat. no.: ab89901), CYP17A1 (Abcam, cat. no.: ab125022), StAR (Abcam, cat. no.: ab96637), PECAM1 (R&D Systems, cat. no.: BBA7), vWF (Abcam, cat. no.: ab6994), CD68 (Santa Cruz Biotechnology, cat. no.: sc-20060), CD11b (Abcam, cat. no.: ab52478), FSH receptor (NOVUS, cat. no.: NBP2-36489), CYP11A1 (Abcam, cat. no.: ab175408), GATA4 (Santa Cruz Biotechnology, cat. no.: sc-25310), and LH receptor (Abcam, cat. no.: ab125214) antibodies. The expression patterns of these proteins were analyzed using fluorescence microscopy (Zeiss LSM 510 Meta).

### Real-time PCR

Total RNA was isolated using TRIzol reagent (Invitrogen Life Technologies) according to the manufacturer’s instructions. The qualities of the purified RNAs were verified by measuring the 260/280 nm absorbance ratio. First-strand cDNA was produced from total RNA (1 μg) using SuperScript II (Invitrogen Life Technologies), and 10% of the total cDNA was used to produce each PCR mixture containing Express SYBR-Green qPCR Supermix (BioPrince, Seoul, South Korea). Real-time PCR was performed using a QIAGEN real-time PCR cycler (Rotor-Gene Q). The reaction was subjected to 40 cycles of amplification at 95 °C for 20 sec, 60 °C for 20 sec, and 72 °C for 25 sec. The relative mRNA expression of the selected genes was normalized to that of PPIA and quantified using the ΔΔCT method. The sequences of the PCR primers used are listed in Table 1.

**Table 1:** Primer sequences for quantitative RT‒PCR.

Gene	Gene bank no.	Direction	Primer sequence
Human PPIA	NM_021130	F	TGCCATCGCCAAGGAGTAG
		R	TGCACAGACGGTCACTCAAA
Human SERPINB2	NM_001143818	F	ACCCCCATGACTCCAGAGAACT
		R	GAGAGCGGAAGGATGAATGGAT
Human StAR	NM_000349	F	CCACTTGCATGGTGCTTCAC
		R	GGGACAGGACCTGGTTGATG
Human NR5A1	NM_004959	F	GCGGGCATGGACTATTCGTA
		R	GAGCAGTCCGTAGTGGTAGC
Human CYP11A1	NM_000781	F	AGCCCACCTTCTTCTTCGAC
		R	TTTGCTCAGCCATCGGGTT
Human HSD17B1	NM_000413	F	CTCGAAGGCTTATGCGAGAGT
		R	ACTCGATCAGGCTCAAGTGG
Human LHR	NM_000233	F	ACCTCCCTGTCAAAGTGATCC
		R	AGGTTGTCAAAGGCATTAGCTTC

### Protein isolation and western blotting analysis

The cells were lysed in a buffer containing 50 mM Tris, 5 mM EDTA, 150 mM NaCl, 1 mM DTT, 0.01% NP 40, and 0.2 mM PMSF. The protein concentrations in the total cell lysates were measured using bovine serum albumin solutions with several concentrations as reference standards. Samples containing equal amounts of protein were separated according to their molecular weight by sodium dodecyl sulfate‒polyacrylamide gel electrophoresis (SDS‒PAGE). The proteins were then transferred electrophoretically to nitrocellulose membranes (Bio-Rad Laboratories). The membranes were blocked with 5% w/v nonfat dry milk in Tris-buffered saline containing Tween-20 for 1 h at room temperature. The membranes were then incubated overnight at 4 °C with the following primary antibodies: anti-SERPINB2 (Abcam, MA, USA, ab47742), anti-MMP-2 (Cell Signaling #4022), anti-MMP-9 (Cell Signaling #13667), anti-caspase-3 (Cell Signaling, MA, USA, #9662), anti-WT1 (Abcam, cat. no.: ab89901), anti-FSH receptor (NOVUS, cat. no.: NBP2-36489), anti-SOX9 (Abcam, cat. no.: ab185230), anti-GATA4 (Santa Cruz Biotechnology, cat. no.: sc-25310), anti-CYP17A1 (Abcam, cat. no.: ab125022), anti-StAR (Abcam, cat. no.: ab96637), anti-LH receptor (Abcam, cat. no.: ab125214), and anti-β-actin (Abcam, MA, USA, ab189073). The membranes were then incubated with HRP-conjugated goat anti-rabbit IgG (BD Pharmingen, San Diego, CA, USA, 554021) and HRP-conjugated goat anti-mouse IgG (BD Pharmingen, 554002) secondary antibodies for 60 min at RT. The antibody-bound proteins were detected using an ECL reagent.

### Transwell migration/invasion assay

Cells (1 × 10^5^ cells/well) were loaded in the upper chambers of a transwell plate (Corning Inc., Corning, NY, USA) to track cell migration through a permeable membrane. The transwell chambers contained 8.0 μm pores in 6.5-mm diameter polycarbonate membranes, and a 24-well plate was used. Noninvading cells on the upper chambers of each membrane were removed by scrubbing them with laboratory paper. The migrated cells on the lower side of each membrane were fixed with 4% paraformaldehyde for five minutes and stained with hematoxylin for 15 minutes. The migrating/invading cells were counted in three randomly selected fields of the wells under an optical microscope at ×50 magnification.

### ELISAs

The levels of ABP (MyBioSource, cat. no.: MBS9310104) and testosterone (R&D Systems, cat. no.: KGE010) secreted by the Sertoli cell- and Leydig cell-loaded chamber, respectively, were analyzed using ELISA kits according to the manufacturer’s instructions. The optical density of each well was then measured with a microplate reader at 450 nm, and the ABP or testosterone concentration in each sample was determined using a standard curve. The experiments were carried out in triplicate. Each sample was analyzed in duplicate.

### SERPINB2 knockdown using specific shRNAs

Small hairpin RNA (shRNA: accession no. NM_002575) targeting SERPINB2 and scrambled shRNA (shCon) were purchased from Bioneer (Daejeon, South Korea). For efficient SERPINB2 transfection, Lipofectamine 2000 (Invitrogen, cat. no.: 52887) was used according to the manufacturer’s instructions. Briefly, shRNA targeting SERPINB2 (3 μg/ml) was mixed with 3 μl of the transfection reagent Lipofectamine 2000 in Gibco Opti-MEM without serum or antibiotics. Five hours before transfection, the Opti-MEM was replaced with fresh Sertoli cell medium supplemented with Sertoli cell growth medium and 10% serum. The SERPINB2 shRNA construct, which is designed from the target sequence and has the most effective transfection efficiency at the mRNA level, was chosen.

### Statistical analysis

All the statistical data were analyzed in GraphPad Prism 9.0 (GraphPad Software, San Diego, CA) and evaluated using two-tailed Student’s *t* tests. *P* values < 0.05 were considered to indicate a significant difference.

## Results

### Synthesis of a testis-on-a-chip platform that properly reflects multicellular complexity and reciprocal endocrine crosstalk

Figure 1a presents the overall concept of the testis-on-a-chip platform, which appropriately reflects the multicellular complexity of various seminiferous tubule constituent cells and their reciprocal endocrine crosstalk. The Sertoli and Leydig cell chambers were interconnected by a vessel channel, which was coated with vascular endothelial cells to allow reciprocal endocrine crosstalk via the exchange of the various reproductive steroids or endocrine factors secreted by these cells. In addition, these two chambers and the connecting vessel channels were surrounded by representative testicular immune cell macrophages to properly mimic their interactions with immune cells within the seminiferous tubules. The testis-on-a-chip development process was performed in several steps. The first step was synthesizing a testis-on-a-chip platform that properly epitomizes the various structural and functional properties of the seminiferous tubules. The chip platform was fabricated by producing a casting mold using a polylactic acid (PLA)-based 3D printing technique. PDMS was then injected into the casting mold for chip fabrication. The oval-shaped testis-on-a-chip platform was separated from the casting mold after polymerization (illustrated in Fig. 1b, c). Subsequently, the cells were mixed with the natural polymer mixture (collagen and hyaluronic acid) and blood coagulation factors (fibrinogen and thrombin) to mimic the 3D tissue microenvironment of the seminiferous tubules and loaded into the corresponding compartments of the chip platform (Fig. 1d).

### Mechanical properties of the natural polymer-based tissue architecture within the human testis-on-a-chip platform

Hyaluronic acid and collagen mixtures have been used in many fields of tissue engineering to mimic the tissue microenvironment composed of extracellular matrix (ECM) owing to their simple structure, relatively high uniformity, and biocompatibility^23–25^. These natural polymers provide a structural tissue framework that supports cell attachment and survival and critical signaling molecules to control various cellular functions, such as cell proliferation, migration, and differentiation^26,27^. Therefore, hybrid biomaterials containing different ratios of collagen and hyaluronic acid were loaded into the fabricated chip platform as scaffolding materials to provide structural support for the attachment of various testicular cells and their subsequent functional integration. However, the mechanical strength of this natural polymer-based tissue architecture was not sufficient to reflect the physical properties of a wide range of normal tissues. Thus, blood coagulation factors (fibrinogen and thrombin) were adopted as natural curing agents to reinforce the mechanical strength without causing cytotoxicity. The fabricated natural polymer-based tissue architecture was white in color and had a soft texture (Supplementary Fig. 1a, b). The physical properties, including the 3D microstructure, mechanical strength, swelling ratio, and rheological behavior (viscosity), were then analyzed. The microstructures of the tissue architecture within the chip platform were analyzed by scanning electron microscopy (SEM). The results revealed uniformly distributed porous microstructures with pore sizes ranging from 50–100 μm throughout the tissue architecture within the chip platform (Fig. 1e). One of the major challenges when designing a natural polymer-based tissue architecture the maintenance of a certain degree of mechanical stability to sustain specific structures and properly support cell survival and the activities of the embedded cells^28,29^. Swelling analysis of the fabricated tissue architectures in both DW and PBS at 37 °C and pH 7.4 indicated that the amount of water absorbed at body temperature did not significantly change their volume or structure (Fig. 1f). The mechanical strength of the fabricated tissue architectures was ~70 kPa, showing similar physical characteristics to those of testicular tissue^30^ (Fig. 1g). Rheological behavior, which critically affects the cell survival rate, is also closely associated with the mechanical properties of the tissue architecture. For example, the structural fidelity of natural polymer-based tissue architectures is commonly poor in cases of lower viscosity because of rapid spread and deformation, but the survival rate of embedded cells is relatively high^31^. Indeed, the rheological properties of tissue architectures are closely related to several cellular functions, such as viability and adhesion^32^. The rheological properties of the fabricated tissue architectures were analyzed by taking the shear stress and dividing it by the shear rate. The viscosity of the natural polymer-based tissue architectures decreased gradually from ~1000 to 0 Pa-sec as the shear rate increased from 1/s to 10/s (Fig. 1h). These results suggest that this fabricated natural polymer-based tissue architecture has suitable mechanical properties for encapsulating various testicular tissue constituent cells.

### Cytocompatibility of the various embedded cells within the fabricated chip platform: cell distribution patterns, survival rates, metabolic activities, and molecular characteristics

Although the fabricated tissue architecture has optimal mechanical properties for cell embedding, several technical challenges still need to be addressed. These include the homogeneous distribution of the embedded cells, maintenance of their original characteristics, and exertion of their proper functions within tissue architectures. In this context, the distribution patterns, survival rates, metabolic activities, and molecular characteristics of various embedded cells were analyzed in this study. Four different seminiferous tubule constituent cells were loaded into each compartment of the chip platform. Human Sertoli and Leydig cells were freshly isolated from the testes of a patient with complete spermatogenesis who underwent bilateral orchiectomy (Supplementary Fig. 2a). The Sertoli cells were characterized morphologically by their spindle-like shape (Supplementary Fig. 2b) and relatively high self-renewal capacity (Supplementary Fig. 2c). In the seminiferous tubules, Sertoli cells can produce and release androgen-binding protein (ABP) in response to testosterone stimulation^33^. Indeed, the isolated Sertoli cells secreted ABP in response to testosterone stimulation in vitro (Supplementary Fig. 2d). Immunostaining (Supplementary Fig. 2e) and western blotting (Supplementary Fig. 2f) also showed that these cells were strongly positive for various known Sertoli cell biomarkers, such as GATA4 (GATA binding protein 4), FSH (follicle-stimulating hormone) receptor, SOX9 (SRY-box transcription factor 9), and WT1 (Wilms’ tumor 1). The Leydig cells were also characterized morphologically by their spindle-like shape (Supplementary Fig. 3a) and relatively high self-renewal capacity (Supplementary Fig. 3b). These cells secrete testicular testosterone in response to luteinizing hormone (LH) stimulation^34^. The isolated Leydig cells secreted testosterone in response to LH stimulation in vitro (Supplementary Fig. 3c). Interestingly, the expression levels of various steroidogenic enzymes (CYP11A1 and HSD17B1), the Leydig cell-specific transcription factor NR5A1, and the gonadotropin receptor (LH receptor), which are involved in steroid hormone synthesis, were significantly greater in Leydig cells, which mainly produce the representative testicular steroid testosterone, than in Sertoli cells (Supplementary Fig. 3d). In addition, the immunostaining (Supplementary Fig. 3e) and western blotting (Supplementary Fig. 3f) results showed that the Leydig cells were strongly positive for various Leydig cell-specific biomarkers, such as CYP11A1, LH receptor, and StAR. Human umbilical vein endothelial cells (HUVECs) were used as the testicular vessel cell model because of their extensive use in many vascular research areas^35^. These cells were characterized by their round or polygonal shape Supplementary Fig. 4a) and positive immunostaining for platelet endothelial cell adhesion molecule (PECAM)-1 and Von Willebrand factor (vWF) (Supplementary Fig. 4b). In addition, tissue-resident macrophages are involved in various physiological functions of the testis, such as steroidogenesis^36^, spermatogonial differentiation^37^, Leydig cell survival^38^, and immune suppression^39^. Therefore, human macrophages were adopted as a testicular tissue-resident immune cell model. These cells were characterized morphologically by their typical round shape (Supplementary Fig. 5a) and positive expression of CD11b and CD68 (Supplementary Fig. 5b).

**Fig. 2:**
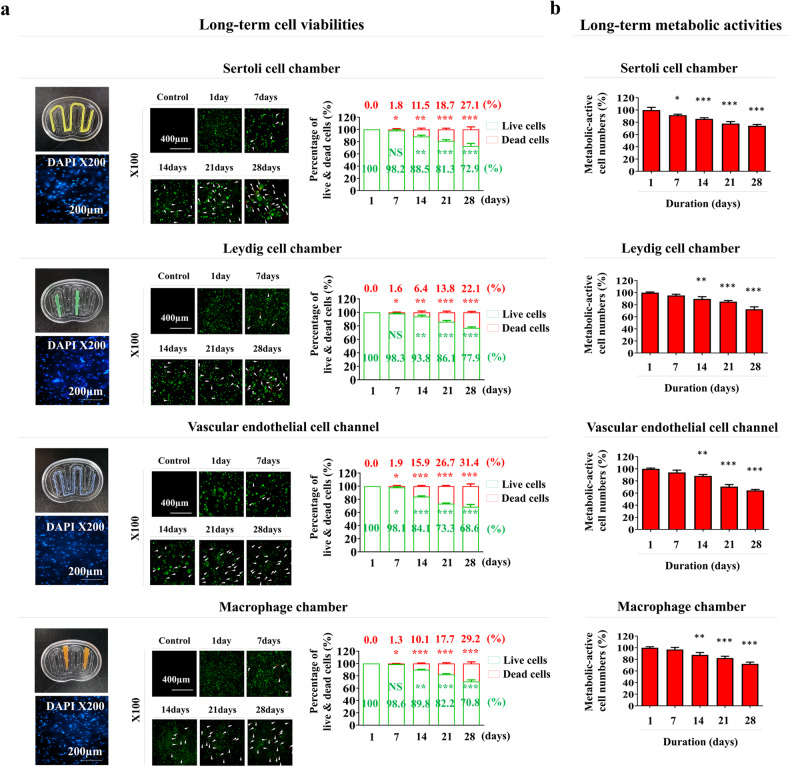
Analysis of the long-term viabilities and metabolic activities of various cells loaded within each chamber of the testis-on-a-chip. To analyze the distribution patterns of various embedded cells within each compartment of the chip, the tissue architectures were incubated with a loaded cell-specific culture medium for 24 h and then with a DNA-specific fluorochrome DAPI labeling solution. The cell distribution patterns within each tissue architecture were determined using a fluorescence microscope. Various loaded testicular tissue constituent cells within each chamber of the testis-on-a-chip, such as Sertoli cells, Leydig cells, vascular endothelial cells, and immune cell macrophages, were stained with DAPI (blue fluorescence) (**a**). Each chamber of the chip was loaded with various types of testicular tissue constituent cells (Sertoli cells, Leydig cells, vascular endothelial cells, and macrophages) and incubated in loaded cell-specific culture medium for one day, seven days, 14 days, 21 days, or 28 days after cell loading. A live/dead assay solution that differentially labels live (green) and apoptotic (red) cells with fluorescent dyes was then added. The long-term cell viability within each chamber was evaluated using a fluorescence microscope. More than 70% of the cells loaded within each chamber of the chip maintained viability after 28 days (apoptotic cells displayed red fluorescence, and living (intact) cells displayed green fluorescence) (**a**). In addition, each chamber of the chip was incubated with CCK-8 reagent in a serum-free environment for 48 h. The metabolic activities of the cells were analyzed by measuring the optical density (OD) at 450 nm (**b**). All experiments were performed in triplicate. Significant differences are indicated as follows: *p < 0.05; **p < 0.005; and ***p < 0.001 (two-sample *t* test).

Next, these seminiferous tubule constituent cells were properly embedded in a natural polymer mixture (collagen and hyaluronic acid) with blood coagulation factors (fibrinogen and thrombin), and their distribution patterns throughout the fabricated tissue architectures were analyzed. It is essential that the loaded cells are homogeneously distributed in the chip platform to achieve a proper tissue microenvironment and obtain accurate assay results^40^. The various embedded cells stained with the nuclear-specific dye DAPI (blue color) were intermixed homogeneously within the fabricated tissue architectures (Fig. 2a). Live/dead cellular assays were also performed at different time points to analyze the long-term survival rates of various embedded cells within the tissue architectures. Although the cell survival rates decreased gradually, most embedded cells (>88%) remained alive after 14 days, and ~70% of the cells were still alive after 28 days (Fig. 2a). In addition, the long-term metabolic activities of various embedded cells within the fabricated tissue architectures were assessed at several time points by performing CCK-8 assays. Although the metabolic activities of various embedded cells gradually decreased with time, ~70% of the cells remained metabolically active after 28 days (Fig. 2b).

**Fig. 3:**
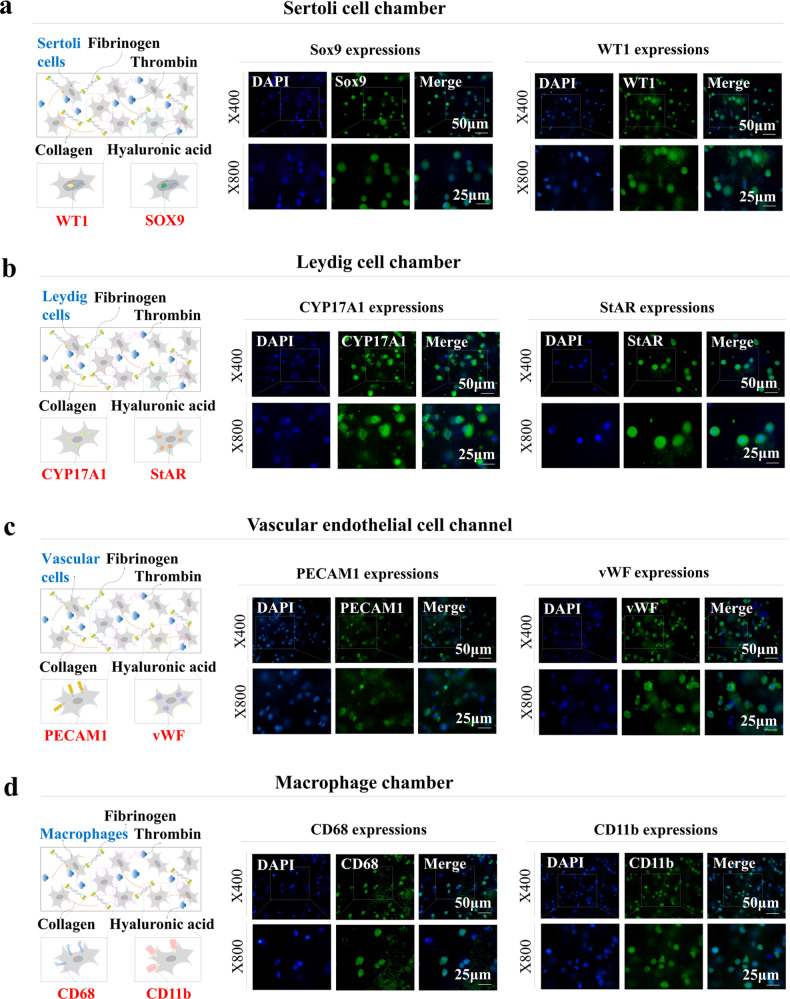
Analysis of the maintenance of cell-specific characteristics within the testis-on-a-chip. To evaluate whether various loaded cells maintained their molecular characteristics after being embedded within each chamber of the chip, the cells were cultured in specific media for one week and analyzed using well-known cell-specific biomarkers. Sertoli cells were stained for WT1 and SOX9 (**a**), Leydig cells were stained for CYP11A1 and StAR (**b**), vascular endothelial cells were stained for PECAM1 and vWF (**c**), and macrophages were stained for CD11b and CD68 (**d**). All experiments were performed in triplicate. DAPI was used to label the nuclei within each field.

Furthermore, various embedded cells were analyzed using specific biomarkers to determine whether these cells can sustain their original molecular properties within the natural polymer-based tissue architecture. Two Sertoli cell markers, SOX9 and WT1, which play key roles in testicular development^41^, were relatively highly expressed in the embedded Sertoli cells (Fig. 3a). CYP11A1 and StAR, which are Leydig cell markers^42^, were expressed in the embedded Leydig cells (Fig. 3b). PECAM1 and vWF, well-known biomarkers for vascular endothelial cells^43^, were expressed in embedded vascular endothelial cells (Fig. 3c). The commonly accepted macrophage biomarkers CD11b and CD68^44^ were also expressed in the encapsulated macrophages (Fig. 3d). These results suggest that multiple types of embedded cells can maintain their original molecular properties within this natural polymer-based tissue architecture.

### Functional analysis of various embedded cells within the chip platform: reproductive hormone secretion and hormone responsiveness

**Fig. 4:**
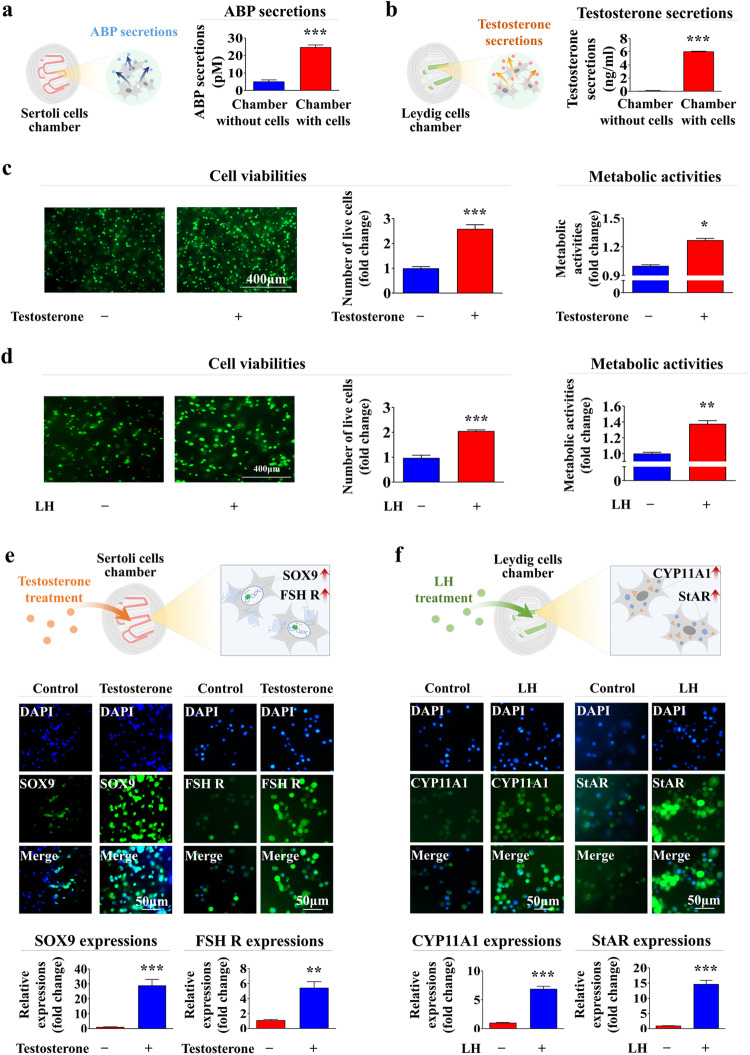
Functional evaluations of the Sertoli and Leydig cell chambers within the chip: endocrine factor secretion, hormone responsiveness, and essential gene expression. A cell-loaded or cell-free chamber was cultured in a specific medium for seven days, after which the medium was changed to serum-free medium to determine whether the Sertoli and Leydig cell chambers of the chip secreted the proper endocrine factors (ABP and testosterone, respectively). After an additional 48 h of incubation, the media were harvested from the Sertoli and Leydig cell chambers, and ABP (**a**) and testosterone (**b**) secretion were analyzed using ELISAs. The Sertoli or Leydig cell chambers were stimulated with or without testosterone or LH, respectively, to determine whether each chamber of the chip could properly respond to endocrine hormones. Subsequently, the cell viabilities and metabolic activities in both the Sertoli (**c**) and Leydig (**d**) cell chambers were determined by performing live/dead and CCK-8 assays. In addition, to assess whether the expression of the specific biomarkers of the loaded Sertoli cells within the chip increased in response to testosterone stimulation, the samples were incubated with or without testosterone for seven days and stained for SOX9 and the FSH receptor (**e**). To determine whether the expression of specific biomarkers of the embedded Leydig cells within the chip could be enhanced in response to LH stimulation, the samples were incubated with or without LH stimulation for seven days. Then, the samples were stained for CYP11A1 and StAR (**f**). All experiments were performed in triplicate. Significant differences are indicated as follows: *p < 0.05, **p < 0.005, and ***p < 0.001 (two-sample *t* test).

In the seminiferous tubules, Sertoli cells secrete an ABP, which binds testosterone and stimulates spermatogenesis and epididymal sperm maturation^33^. Leydig cells in the testis are the primary source of testosterone, which is essential for spermatogenesis and male reproductive development^40^. Thus, this study analyzed whether ABP or testosterone is actively secreted from the Sertoli or Leydig cell chamber using ELISAs. Indeed, ABP and testosterone were produced and secreted from the Sertoli and Leydig cell chambers, respectively (Fig. 4a, b). Responsiveness to steroid hormones or endocrine factors is another important factor in determining whether the testis-on-a-chip functions properly. Therefore, the responsiveness of the Sertoli or Leydig cell chambers to testosterone or LH stimulation was analyzed by assessing cell viability and metabolic activity using live/dead or CCK-8 assays, respectively. Indeed, the cell viability and metabolic activity within the Sertoli and Leydig cell chambers increased significantly by testosterone (Fig. 4c) and LH (Fig. 4d) stimulation, respectively. In addition, the expression of the well-known Sertoli cell markers SOX9 and the FSH receptor in the embedded Sertoli cells increased significantly in response to testosterone stimulation (Fig. 4e). Consistently, the expression of Leydig cell markers, such as CYP11A1 and StAR, was also significantly increased in response to LH stimulation (Fig. 4f). These results suggest that the testis-on-a-chip properly exerts normal physiological functions by secreting various reproductive factors and responding to endocrine hormones. In addition, the effects of the bidirectional cross-communication between the Sertoli and Leydig cell chambers on the functions of the cells loaded in each chamber, especially their viability, were evaluated by performing live/dead assays. Interestingly, in the coculture system with bidirectional cross-communication between the Sertoli and Leydig cell chambers, the cell survival rates were clearly greater than those in the chambers that were cultured separately (Supplementary Fig. 6a, b). The results clearly demonstrated the existence of reciprocal endocrine cross-communication between the Sertoli cell chamber and the Leydig cell chamber.

### SERPINB2 is a reliable biomarker for predicting male reproductive toxicity in human Sertoli and Leydig cells

**Fig. 5:**
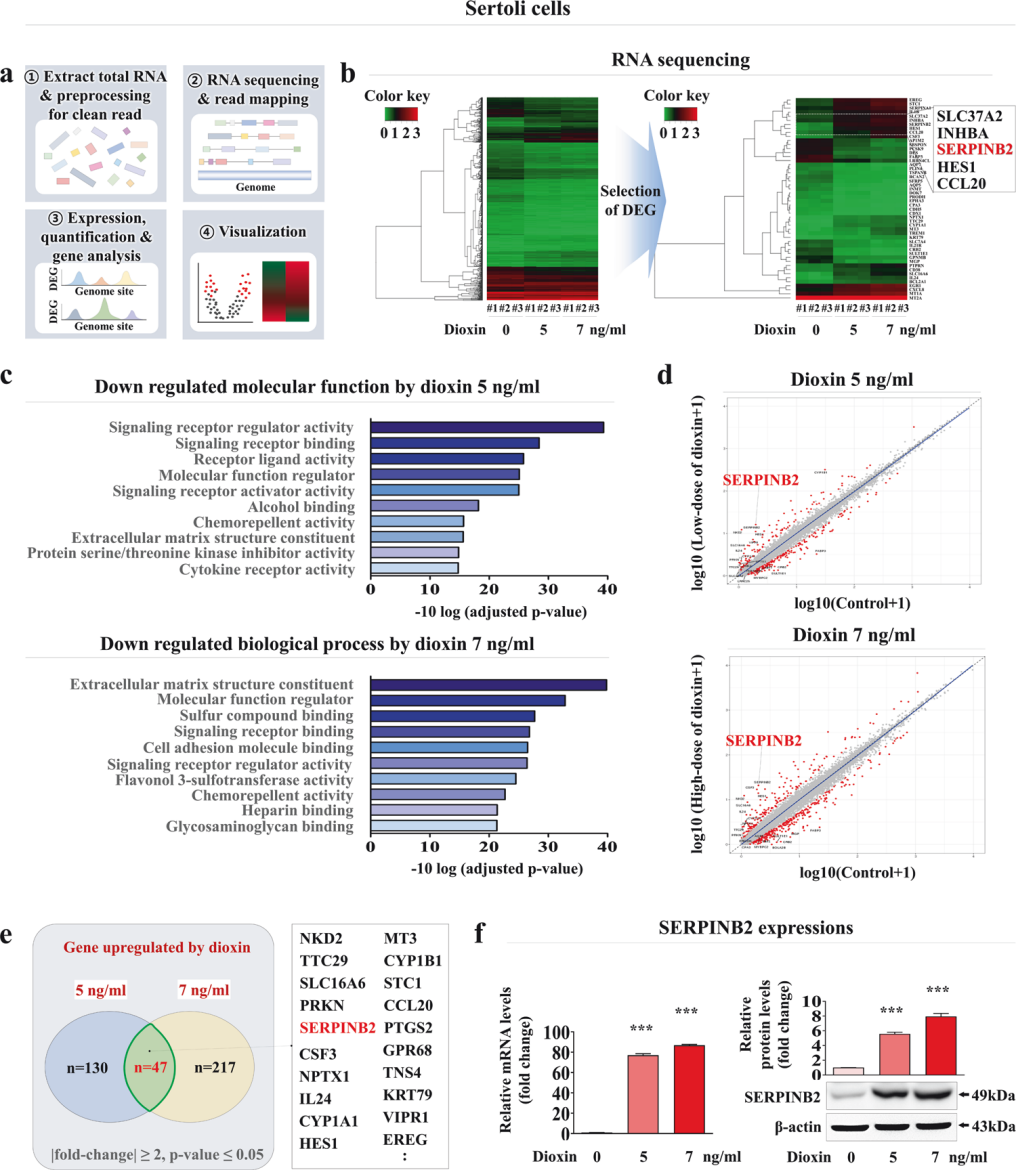
Identification and verification of reliable biomarkers to predict male reproductive toxicity in human Sertoli cells. The major steps for the RNA-sequencing protocol, including the experimental design, read alignment, quantification, and visualization, are presented as a schematic (**a**). The large-scale RNA sequencing data are shown as a heatmap of differential gene expression in the control groups compared to those treated with low (5 ng/ml) and high (7 ng/ml) concentrations of dioxin; increased (red) or decreased (green) gene expression compared to the mRNA levels in the control groups is indicated (**b**). KEGG pathway analysis was subsequently conducted to determine which pathways and functions are likely to be associated with toxin exposure (**c**). Among the differentially expressed genes, a positive correlation was found between the significantly increased SERPINB2 expression levels and toxin exposure in human Sertoli cells (**d**, **e**). Real-time PCR and western blotting were used to verify that the levels of SERPINB2 increased after treatment with low and high concentrations of toxin (**f**). β-actin was used as the internal protein control, and PPIA was used as the housekeeping gene for real-time PCR. All experiments were performed in triplicate. The data are presented as the means ± SDs. *p < 0.05; **p < 0.005; and ***p < 0.001 (two-sample *t* test).

**Fig. 6:**
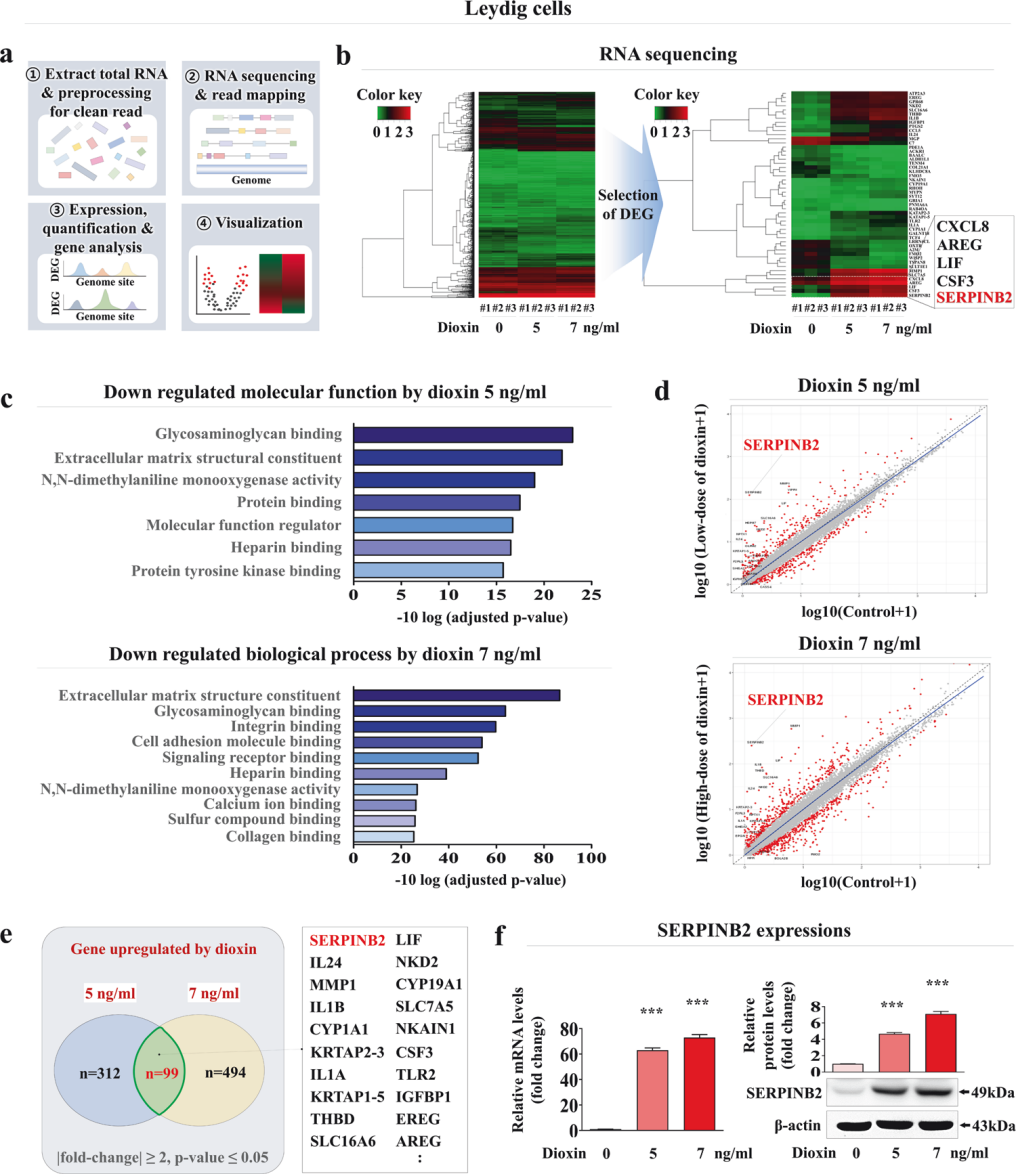
Identification and verification of reliable biomarkers to predict male reproductive toxicity in human Leydig cells. The major steps for the RNA-sequencing protocol, including the experimental design, read alignment, quantification, and visualization, are presented as a schematic (**a**). The large-scale RNA sequencing data are shown as a heatmap of differential gene expression in the control groups compared to those treated with low (5 ng/ml) and high (7 ng/ml) concentrations of dioxin; increased (red) or decreased (green) gene expression compared to the mRNA levels in the control groups is indicated (**b**). KEGG pathway analysis was subsequently conducted to determine which pathways and functions are likely to be associated with toxin exposure (**c**). Among the differentially expressed genes, a positive correlation was found between significantly increased SERPINB2 levels and toxin exposure in human Leydig cells (**d**, **e**). Real-time PCR and western blotting were used to verify that the levels of SERPINB2 increased after treatment with low and high concentrations of toxin (**f**). β-Actin was used as the internal protein control, and PPIA was used as the housekeeping gene for real-time PCR. All experiments were performed in triplicate. The data are presented as the means ± SDs. *p < 0.05; **p < 0.005; and ***p < 0.001 (two-sample *t* test).

To develop a biomarker-based screening platform for male reproductive toxicity, a reliable biomarker was first identified by analyzing large-scale gene expression patterns in human Sertoli and Leydig cells exposed to a toxin via RNA sequencing. Figures 5a and 6a present a schematic summary of multiple RNA sequencing analysis steps in both cell types. Dioxin (TCDD), from the list of top-ranked chemicals based on the hazards of five different international authorities, was selected as a reference toxic material^45^. Several genes whose expression increased markedly in response to toxin exposure were identified in both cell types (Figs. 5b and 6b). KEGG pathway analysis was also performed to predict the functional interactions between the toxin and various signaling networks. Multiple toxicity-associated signaling networks were markedly activated in response to toxin exposure in both cell types (Figs. 5c and 6c). Among the genes whose expression increased in response to toxin exposure in the low and high-concentration treatment groups, a significant positive correlation was observed between increased SERPINB2 expression and toxin exposure in both cell types (Figs. 5d, e and 6d, e). These results were then verified using real-time PCR and western blotting in Sertoli (Fig. 5f) and Leydig (Fig. 6f) cells.

Apoptosis and SERPINB2-associated signaling networks were analyzed using ingenuity pathway analysis (IPA) to determine whether the activation of SERPINB2-associated signaling networks was positively correlated with toxin responses, such as growth arrest and apoptotic cell death. Interestingly, positive regulators of SERPINB2, such as IL-1R and NF-κB, were activated in dioxin-treated Sertoli cells (Supplementary Fig. 7a). Consistently, positive regulators of SERPINB2 (NF-κB and TP63) were also activated in dioxin-treated Leydig cells (Supplementary Fig. 7b). Moreover, the Gene Expression Omnibus (GEO) database was also used to confirm the positive correlation between increased SERPINB2 expression and exposure to multiple toxins, such as dioxin, formaldehyde, nickel, silicon dioxide, and benzene (Supplementary Fig. 8). In addition, Sertoli (Supplementary Fig. 9) and Leydig (Supplementary Fig. 10) cells were treated with multiple types of well-known toxicants, and then, the mRNA levels of the identified reproductive toxicity marker SERPINB2 in cells were evaluated using real-time PCR to determine whether SERPINB2 can be a universal biomarker for other toxic substances in addition to dioxin.

Next, SERPINB2 expression was knocked down using a specific shRNA targeting SERPINB2 in Sertoli (Supplementary Fig. 11a–c) and Leydig (Supplementary Fig. 12a–c) cells to verify that SERPINB2 functions as a critical regulator of various toxicity-associated cellular events, such as apoptosis, growth, and migration. The effects on various cellular functions with or without dioxin exposure were then investigated (Supplementary Fig. 13a and Supplementary Fig. 14a). Importantly, the toxicant-induced suppression of cell growth was abolished by SERPINB2 knockdown in Sertoli (Supplementary Fig. 13b) and Leydig (Supplementary Fig. 14b) cells. The toxicant-induced inhibitory effects on cell migration/invasion (Supplementary Fig. 13c and Supplementary Fig. 14c) and MMP-2/9 expression (Supplementary Fig. 13d and Supplementary Fig. 14d) were also significantly attenuated by SERPINB2 depletion in both cell types. In addition, the toxicant-induced stimulating effects on apoptotic DNA fragmentation (Supplementary Fig. 13e and Supplementary Fig. 14e) and proapoptotic caspase-3 activities (Supplementary Fig. 13f and Supplementary Fig. 14f) were also attenuated by SERPINB2 depletion in both cell types.

### Development of a fluorescent protein-conjugated toxicity marker reporter system to quantify male reproductive toxicity

**Fig. 7:**
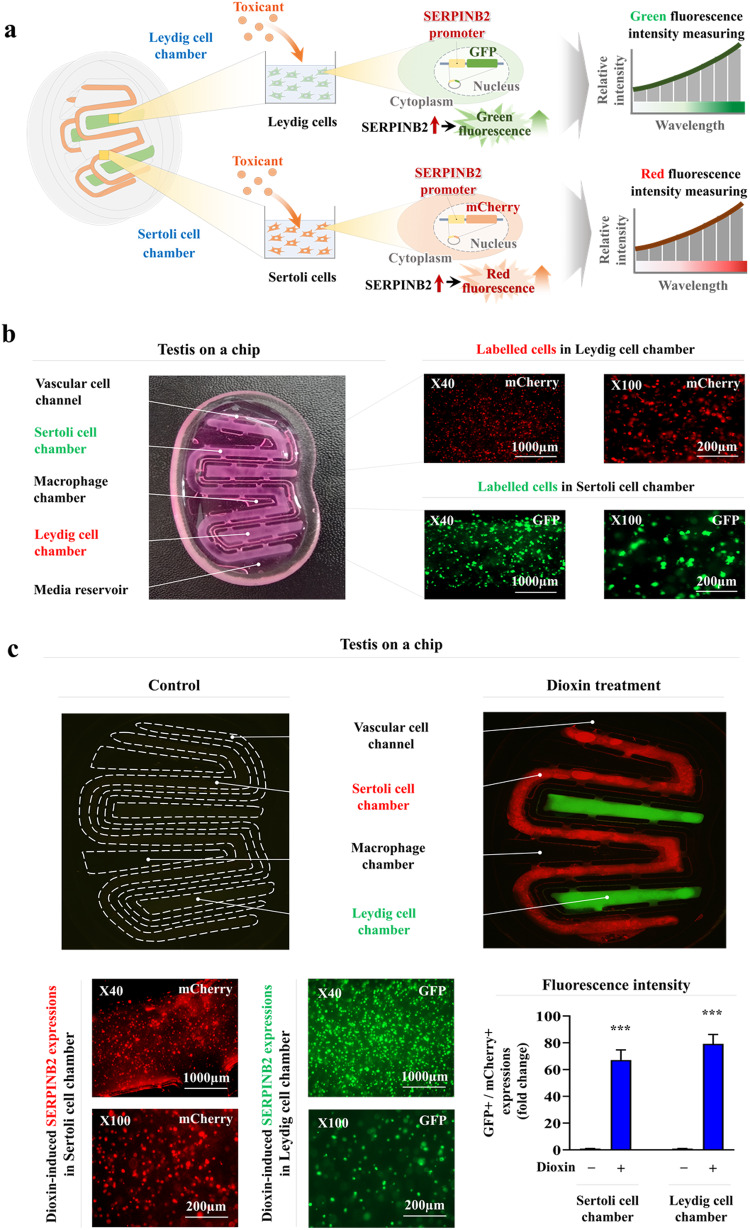
Establishment of toxicity biomarker-conjugated fluorescent reporter system in both the Sertoli and Leydig chambers of the testis-on-a-chip. A SERPINB2-conjugated fluorescent reporter system was successfully introduced into Sertoli and Leydig cells so that toxicant-induced SERPINB2 activity could be converted to red (mCherry) or green (GFP) fluorescence signals. Therefore, the male reproductive toxicity of certain drug candidates can be evaluated intuitively and quantitatively by measuring the fluorescence intensity (**a**). This fluorescent reporter system was introduced to the Sertoli or Leydig cells that had been properly loaded into the corresponding chambers of the testis-on-a-chip. The distribution patterns of the loaded cells within each chamber were assessed using a fluorescence microscope (**b**). Toxicant exposure (5 ng/ml dioxin) significantly increased SERPINB2 activity, which was subsequently converted into red or green fluorescence signals in the Sertoli or Leydig cell chambers of the chip, respectively (**c**). The data are presented as the means ± standard deviations. *p < 0.05; **p < 0.005; and ***p < 0.001 (two-sample *t* test).

**Fig. 8:**
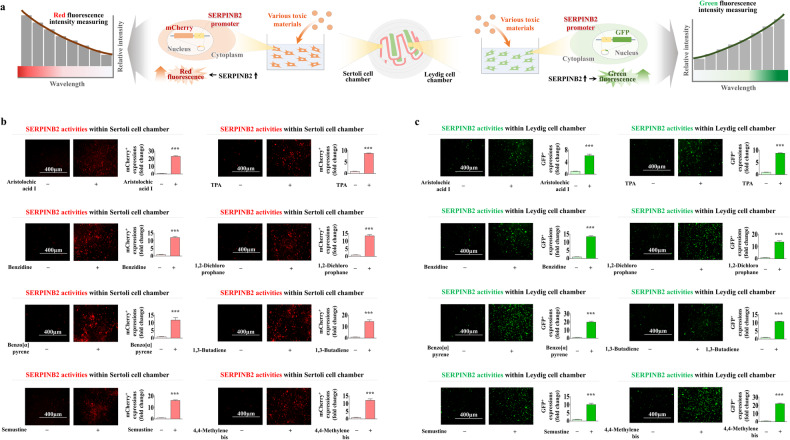
Exposure to various types of toxins induced SERPINB2 activation, which was converted to a red fluorescence signal in the Sertoli cell chamber of the chip. A SERPINB2-conjugated mCherry (red) fluorescent reporter system was successfully introduced into both Sertoli and Leydig cells. These cells were then loaded into the corresponding chamber of the chip (**a**). To verify the reliability of the toxicity biomarker (SERPINB2)-conjugated fluorescent reporter system, the Sertoli cell chamber of the chip was exposed to multiple types of toxicants, including aristolochic acid I (10 μM), benzidine (10 μM), benzo[a]pyrene (2 μM), semustine (0.5 mM), TPA (5 nM), 1,2-dichloropropane (100 mM), 1,3-butadiene (10 mM), and 4,4’-methylenebis (5 μM). SERPINB2 activity in response to toxin exposure was evaluated intuitively and quantitatively by measuring the converted fluorescence intensity (**b**). To verify the reliability of the toxicity biomarker (SERPINB2)-conjugated fluorescent reporter system, the Leydig cell chamber of the chip was exposed to multiple types of toxicants, including aristolochic acid I (10 μM), benzidine (10 μM), benzo[a]pyrene (2 μM), semustine (0.5 mM), TPA (5 nM), 1,2-dichloropropane (100 mM), 1,3-butadiene (10 mM), and 4,4’-methylenebis (5 μM). SERPINB2 activity in response to toxin exposure was intuitively and quantitatively evaluated by measuring the converted fluorescence intensity (**c**). Significant differences are indicated as follows: *p < 0.05; **p < 0.005; and ***p < 0.001 (two-sample *t* test).

Fluorescent gene-based reporter systems are widely used to evaluate the activities of certain genes or signaling pathways and provide a powerful predictive platform for analyzing the therapeutic effects and potential toxicities of drug candidates^46–48^. The results here indicate that SERPINB2 is a reliable and universal biomarker for predicting reproductive toxicity in males. Therefore, a fluorescent protein (mCherry or GFP) was conjugated to the promoter region of SERPINB2 in both Sertoli (Supplementary Fig. 15a) and Leydig (Supplementary Fig. 15b) cells so that toxin exposure can increase SERPINB2 activity, which induces a red or green fluorescence signal in the chip. This system made the intuitive and quantitative assessment of male reproductive toxicity possible by measuring the fluorescence intensity (Fig. 7a). Subsequently, the Sertoli and Leydig cells transfected with a fluorescent protein-conjugated SERPINB2 reporter vector were loaded into the corresponding chambers of the testis-on-a-chip. The embedded Sertoli (labeled with red mCherry) and Leydig (labeled with green GFP) cells were distributed homogenously throughout each chamber of the testis-on-a-chip (Fig. 7b). In addition, the activity of SERPINB2 in response to toxin exposure (dioxin) in both the Sertoli and Leydig cell chambers was evaluated by measuring the fluorescence intensity to confirm the ability of the fluorescent protein-conjugated toxic marker (SERPINB2) reporter platform to detect male reproductive toxicity. Importantly, toxin exposure significantly increased the SERPINB2-conjugated red (mCherry) or green (GFP) fluorescence signal in both chambers of the testis-on-a-chip (Fig. 7c). The activated SERPINB2-induced fluorescence intensity was then analyzed in response to exposure to various toxic materials in the Sertoli and Leydig cell chambers to confirm whether this reporter system could be used as a universal male reproductive toxicity detection platform as opposed to being a specific gene that responds to the selected toxicant dioxin (Fig. 8a). Importantly, all the analyzed toxicants strongly stimulated SERPINB2 activity in the Sertoli (Fig. 8b) and Leydig (Fig. 8c) cell chambers. These results suggest that this fluorescent protein-conjugated toxic marker reporter system is a reliable platform for assessing the potential male reproductive toxicity of certain substances or drug candidates.

## Discussion

Conventional single-cell-based 2D cultures are the most frequently used in vitro assay platforms for analyzing the efficacy or toxicity of potential drug candidates. While these traditional culture models have the advantages of speed, convenience, and scalability, they do not adequately epitomize the complexity of the tissue microenvironment and lack various cell–ECM or cell‒cell communications that commonly occur in tissues^49^. Thus, these cultures sometimes give misleading and unpredictable results regarding in vivo responses^50^. Recent attempts to replicate the complex architecture and functionalities of the testis with organoid models have encountered substantial challenges^51,52^. These include difficulties in precisely representing the multicellular intricacy, three-dimensional context, and the complex hormonal interplay occurring within the testis. Indeed, most current in vitro male reproductive tissue assay systems fail to mimic the complex multicellularity and endocrine crosstalk. For example, the seminiferous tubules of the testis contain multiple intercommunicating cell types, such as Sertoli cells, Leydig cells, immune cells, and vascular endothelial cells^53^. Thus, we have innovated a pioneering 3D culture system known as testis-on-a-chip. This novel platform combines different types of cells found in the testis with biodegradable organic polymers, such as collagen and hyaluronic acid. This fused system effectively recreates the distinctive attributes and intricacies of testicular tissue, allowing for an accurate simulation of the multicellular characteristics of complex tissues.

Similarly, various recent organ-on-chip platforms have provided promising alternatives for mimicking the dynamic and multicellular in vivo niche microenvironment of a male reproductive organ. For example, Komeya et al. embedded mouse testicular tissues into an agarose gel block and placed it on a nonfluidic PDMS ceiling chip^15^. After several weeks of culture, spermatogenesis was evaluated in vitro by analyzing meiotic germ cell numbers. Sharma et al. also developed an ex vivo culture system for human and nonhuman primate seminiferous tubule tissues in a PDMS-based microfluidic chip platform without cell isolation processes^12^. Similarly, Komeya et al. introduced a mouse testicular tissue fragment-loaded microfluidic device with a porous membrane to separate each chamber^13^. This ex vivo mouse testicular tissue fragment culture platform supported spermatogenesis and testosterone secretion for up to 24 weeks. Although these whole-tissue ex vivo culture platforms can adequately mimic the physiological and structural features of complex organs or tissues composed of multiple cell types, several limitations, such as their high cost and variability with poor reproducibility, must be overcome.

Therefore, Abu Madighem et al.^11^ developed a microfluidic testis chip device using testicular cells derived from mouse seminiferous tubules. These mouse testicular cells can develop into spheroids. The cells were embedded in methylcellulose gel and cultured in a microfluidic chip device. Although these embedded spheroids on the chip platform exhibited testosterone responsiveness and the ability to differentiate into early-stage germ cells, they did not adequately mimic the physiological features of human seminiferous tubules because it is a mouse cell-based system. Interestingly, Baert et al. developed a chip-based in vitro coculture model to mimic the systemic interaction between the liver and testis using human liver and testicular spheroids^16^. Unfortunately, these spheroid-based chip platforms did not properly reflect the reciprocal endocrine crosstalk between the Sertoli and Leydig cells within seminiferous tubules or their complex structural features because they were fabricated as a spheroid-embedded chip platform without a specific testicular structure.

Therefore, a novel human testis-on-a-chip platform was developed to mimic the reciprocal endocrine crosstalk between the Sertoli and Leydig cells in seminiferous tubules within a highly compartmentalized multicellular chip platform. Indeed, testosterone and the male endocrine factor ABP, which can control various essential functions of testicular tissue and subsequent spermatogenesis, were produced in and released from the fabricated testis-on-a-chip (Fig. 4a, b). In addition to Sertoli and Leydig cells, other cellular components of human testicular tissue, such as human immune cells (macrophages) and vascular endothelial cells, were integrated into the chip platform with a natural polymer mixture (collagen and hyaluronic acid) and blood coagulation factors (fibrinogen and thrombin).

Another critical challenge to be addressed in the successful development of testis-on-a-chip is the achievement of optimal culture conditions that can maintain the various physiological properties of multiple embedded cell combinations. Because each embedded cell in the chip platform is subjected to its optimal culture conditions, various cell culture media were mixed at a certain ratio (Sertoli cell medium:EGM-2:PMI1640 = 1:1:1) to maintain their specific characteristics within the chip. Indeed, the long-term survival rates (Fig. 2a) and metabolic activities (Fig. 2b) of these cells were relatively well maintained in the testis-on-a-chip. In addition, whether various testicular cells embedded within the chip platform could properly sustain their specific biomarker expression in each compartment on a chip was evaluated (Fig. 3a–d).

Specific toxicity biomarker-based screening platforms can provide more rapid and sensitive information on certain unknown materials than can phenotypic or functional parameters (cell proliferation or apoptotic cell death)-based platforms^20^. Thus, RNA-sequencing analysis after exposure to low and high concentrations of toxicants was performed to identify a reliable biomarker for predicting male reproductive toxicity. A small subset of genes closely associated with the toxicity phenotypes in human Sertoli (Fig. 5) and Leydig (Fig. 6) cells was observed. One of the genes identified, SERPINB2, also known as PAI-2, was significantly activated in both testicular cell types in response to various types of toxicants (Supplementary Figs. 8 and 9). Consistent with these findings, previous studies revealed that SERPINB2 activity was significantly increased in human endometrial stem cells^54^, umbilical cord blood stem cells^45^, and multiple types of cancer stem cells^55^ in response to exposure to various toxicants. Importantly, the toxicant-induced inhibitory effects on various cellular functions were significantly abolished by SERPINB2 knockdown in Sertoli (Supplementary Fig. 12) and Leydig (Supplementary Fig. 13) cells. These results suggest that SERPINB2 is a reliable biomarker of male reproductive toxicity. Subsequently, a fluorescent gene (red, mCherry; or green, GFP) was conjugated to the promoter region of SERPINB2 in Sertoli (Supplementary Fig. 14a) and Leydig (Supplementary Fig. 14b) cells and the activity of the reporter in response to toxin exposure was evaluated quantitatively by measuring its fluorescence intensity (Fig. 7). Furthermore, significantly increased SERPINB2-mediated fluorescence (GFP or mCherry) was observed in both Sertoli (Fig. 8b) and Leydig (Fig. 8c) cells after exposure to multiple types of toxicants. These findings illuminate the critical requirements for developing a biomarker-based screening platform to evaluate the male reproductive toxicity of potential drug candidates.

### Supplementary information

Supplementary information
