# Supplementary material for: Development of a novel testis-on-a-chip that demonstrates reciprocal crosstalk between Sertoli and Leydig cells in testicular tissue
Source: Exp Mol Med. 2024 Jul 1;56(7):1591–605. doi: 10.1038/s12276-024-01258-3 (PMC11297247; doi:10.1038/s12276-024-01258-3)
Supplement: Supplementary file 1 — Supplementary information [file 12276_2024_1258_MOESM1_ESM.pdf]

Supplementary figure legends

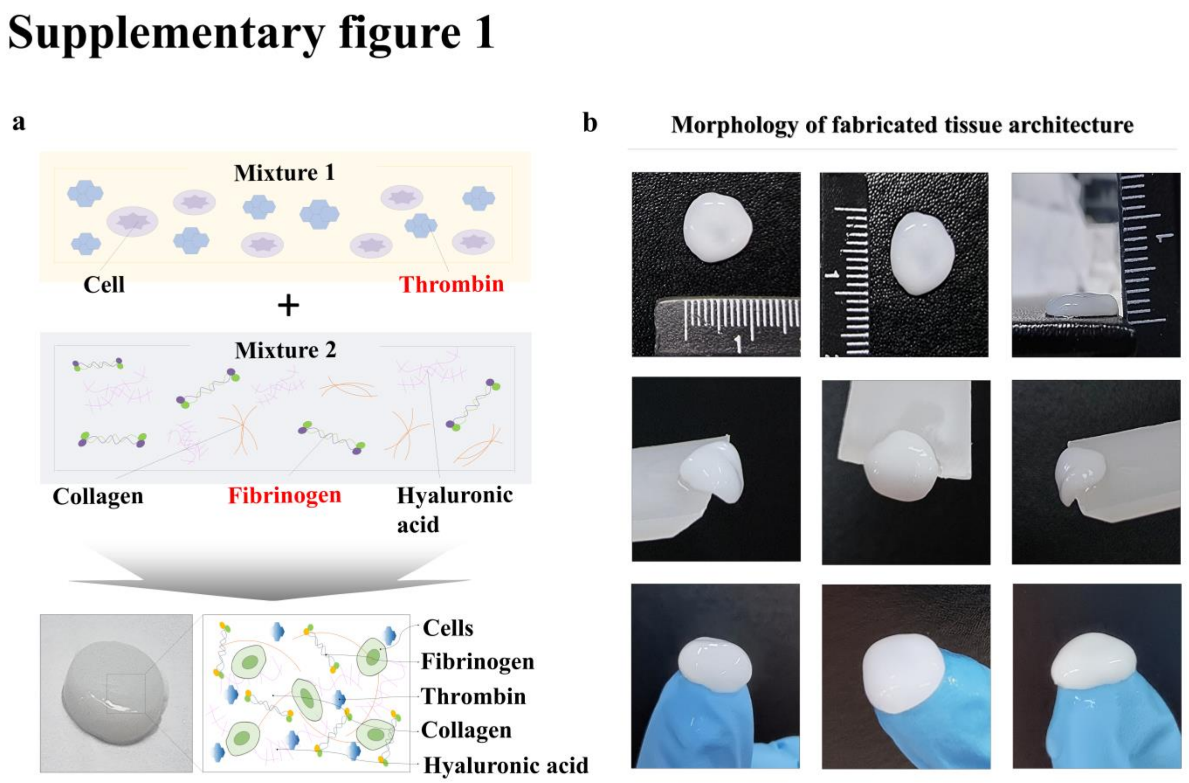

Supplementary figure 1. The fabrication natural polymer-based tissue architecture by incorporating blood coagulation factors. Insufficient mechanical strength of the natural polymer mixture (collagen and hyaluronic acid) was properly reinforced by incorporating blood coagulation factors (fibrinogen and thrombin) as a non-toxic cross-linker agent **(a)**. The fabricated natural polymers-based tissue architecture, encapsulating various testicular cellular components, showed white color with a very soft texture **(b)**.

Supplementary figure 2

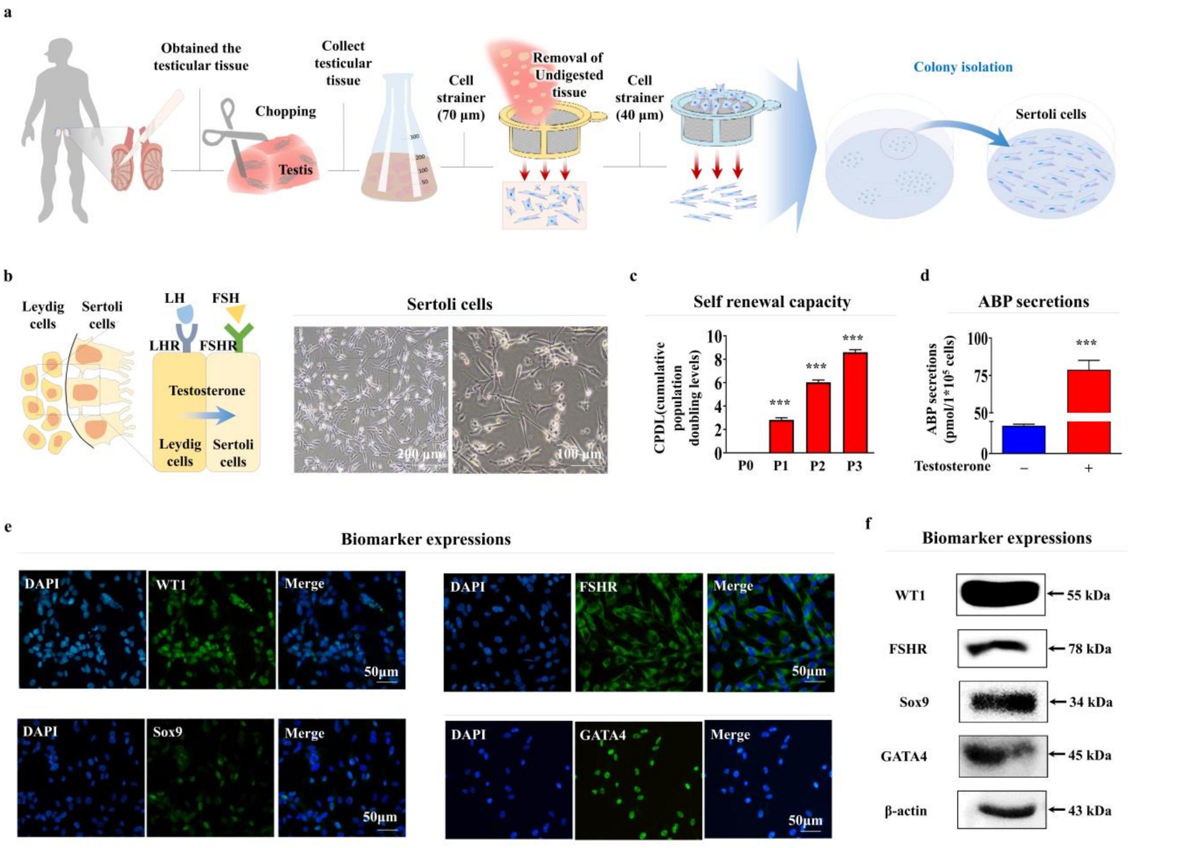

Supplementary figure 2. Isolation and characterization of human testicular Sertoli cells. Schematic representation showing the isolation procedures for testicular Sertoli cells from a patient with complete spermatogenesis undergoing bilateral orchiectomy. Sertoli cells in human testicular tissue biopsy were isolated from other cell types by filtering through the cell strainers with various pore sizes **(a)**. Spindle-shaped human Sertoli cells were observed by phase-contrast microscopy **(b)**. The proliferative capacity of isolated Sertoli cells was determined by measuring the cumulative population doubling levels between passages 0 and 4 **(c)**. This study assessed whether androgen-binding protein (ABP) was produced and secreted from isolated Sertoli cells in response to testosterone stimulation using ELISA **(d)**. The expression levels of well-known Sertoli cell markers, such as GATA4, FSH receptor (FSHR), SOX9, and WT1, were evaluated by immunostaining **(e)**. The expression levels of GATA4, FSHR, SOX9, and WT1 in isolated Sertoli cells were also evaluated by western blotting **(f)**. β-actin was used as the internal control. Significant differences are presented. *$p < 0.05$, **$p < 0.005$, and ***$p < 0.001$ (two-sample t-test).

Supplementary figure 3

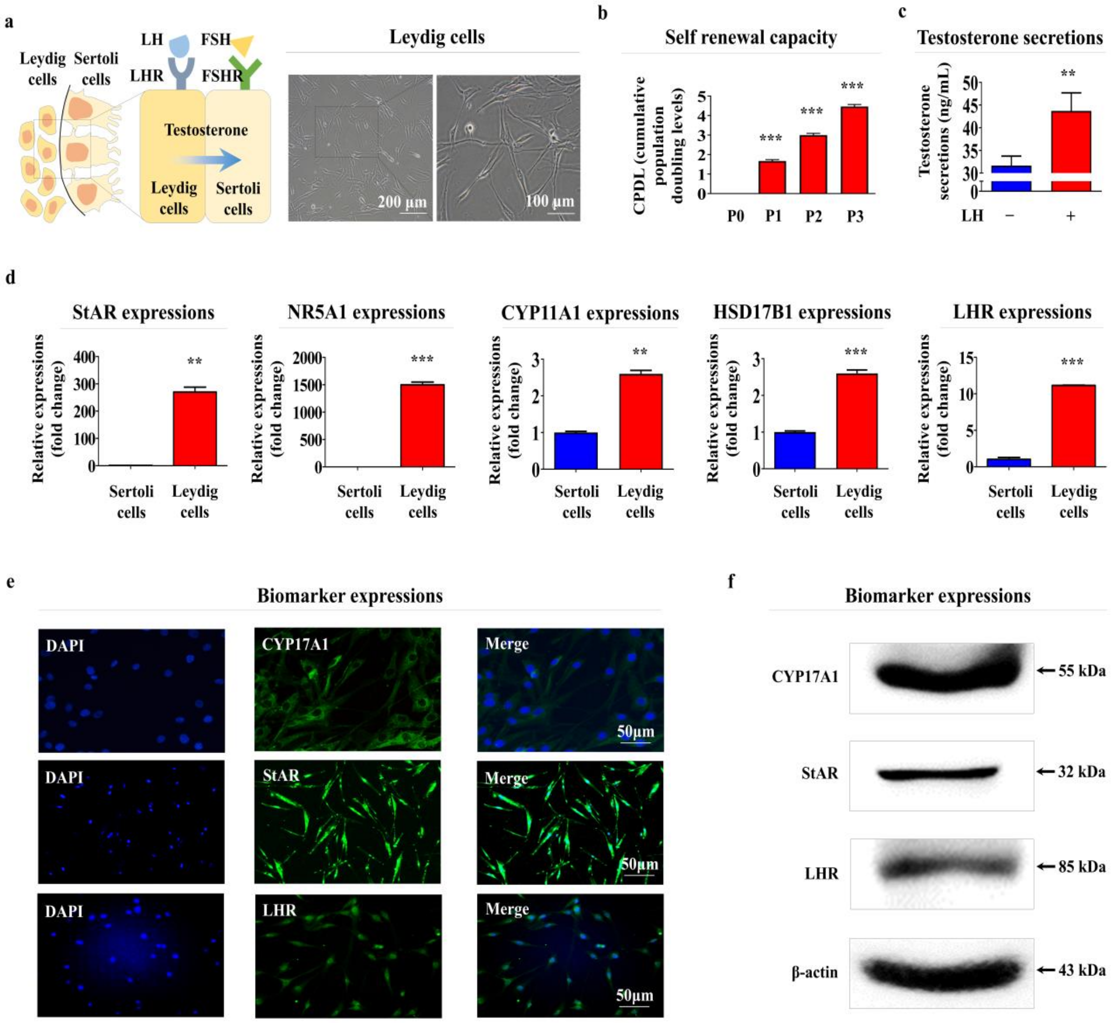

Supplementary figure 3. Isolation and characterization of human testicular Leydig cells. Leydig cells in human testicular tissue biopsy were isolated from other cell types by filtering through the cell strainers with various pore sizes Spindle-shaped human Leydig cells were observed by phase-contrast microscopy **(a)**. The proliferative capacity of isolated Leydig cells was determined by measuring the cumulative population doubling level between passages 0 and 3 **(b)**. This study assessed whether testosterone was produced and secreted from the isolated Leydig cells in response to LH stimulation using ELISA **(c)**. The mRNA levels of steroidogenic enzymes (CYP11A1, HSD17B1, and StAR), Leydig cell-specific transcription factor (NR5A1), and gonadotropin receptor (LH receptor) were compared in both Sertoli and Leydig cells by real-time PCR **(d)**. The protein levels of CYP17A1, LH receptor (LHR), and StAR were evaluated by immunostaining **(e)**. The expression patterns of CYP17A1,

LHR, and StAR in isolated Leydig cells were also further evaluated by western blotting **(f)**. β-actin was used as the internal protein control and PPIA as the housekeeping gene for real-time PCR. Significant differences are presented. *$p < 0.05$, **$p < 0.005$, and ***$p < 0.001$ (two-sample t-test).

Supplementary figure 4

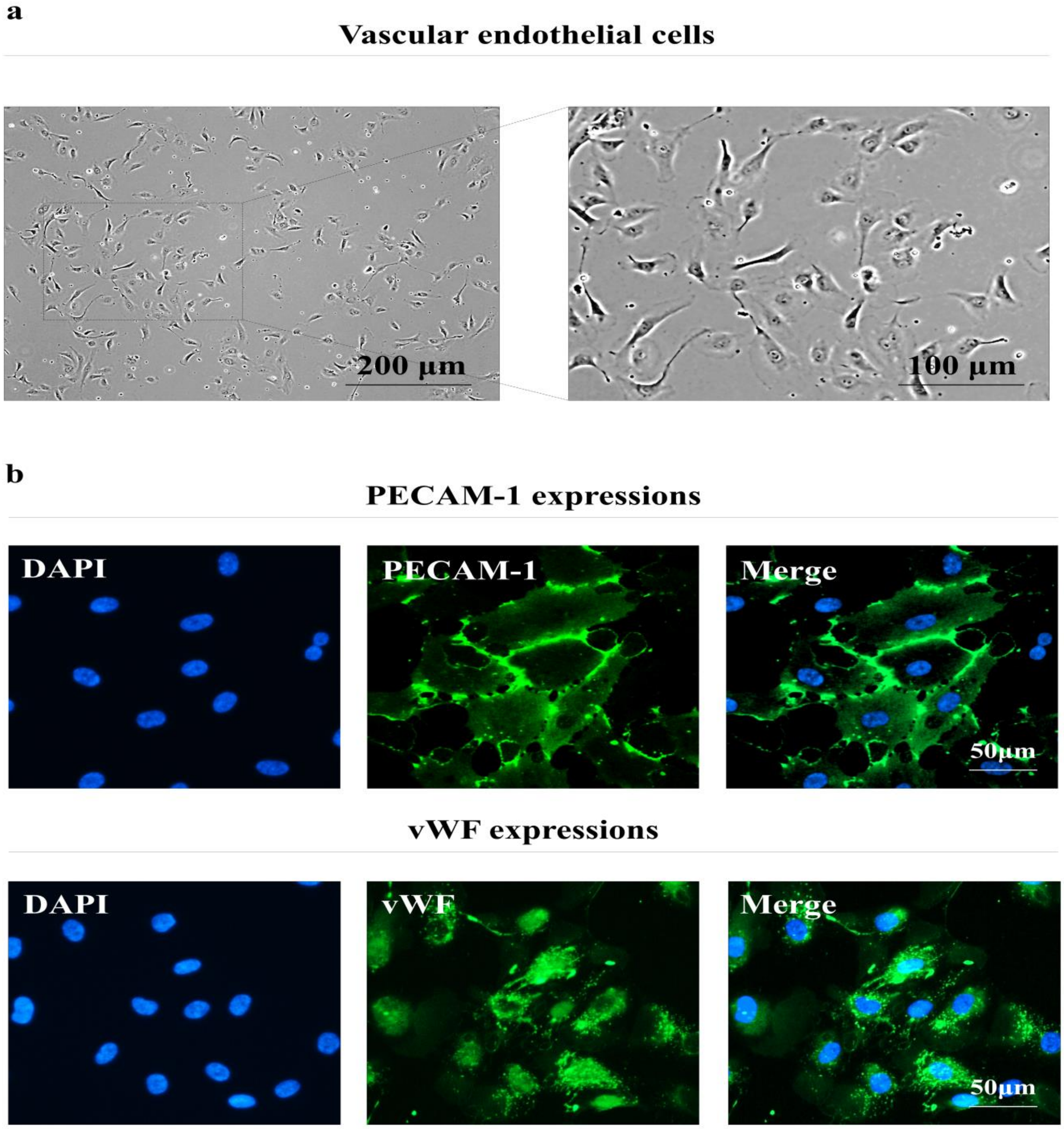

Supplementary Figure 4. Molecular characterization of human vascular endothelial cells. Human umbilical vein endothelial cells (HUVECs) were grown as a monolayer culture. Their characteristic polygonal shape was observed by phase-contrast microscopy **(a)**. Expression patterns of putative markers platelet endothelial cell adhesion molecule-1 (PECAM-1) and von Willebrand Factor **(**vWF) as putative endothelial cell-specific markers were analyzed by immunofluorescent staining **(b)**. DAPI staining was used to label the nuclei within each field.

Supplementary figure 5

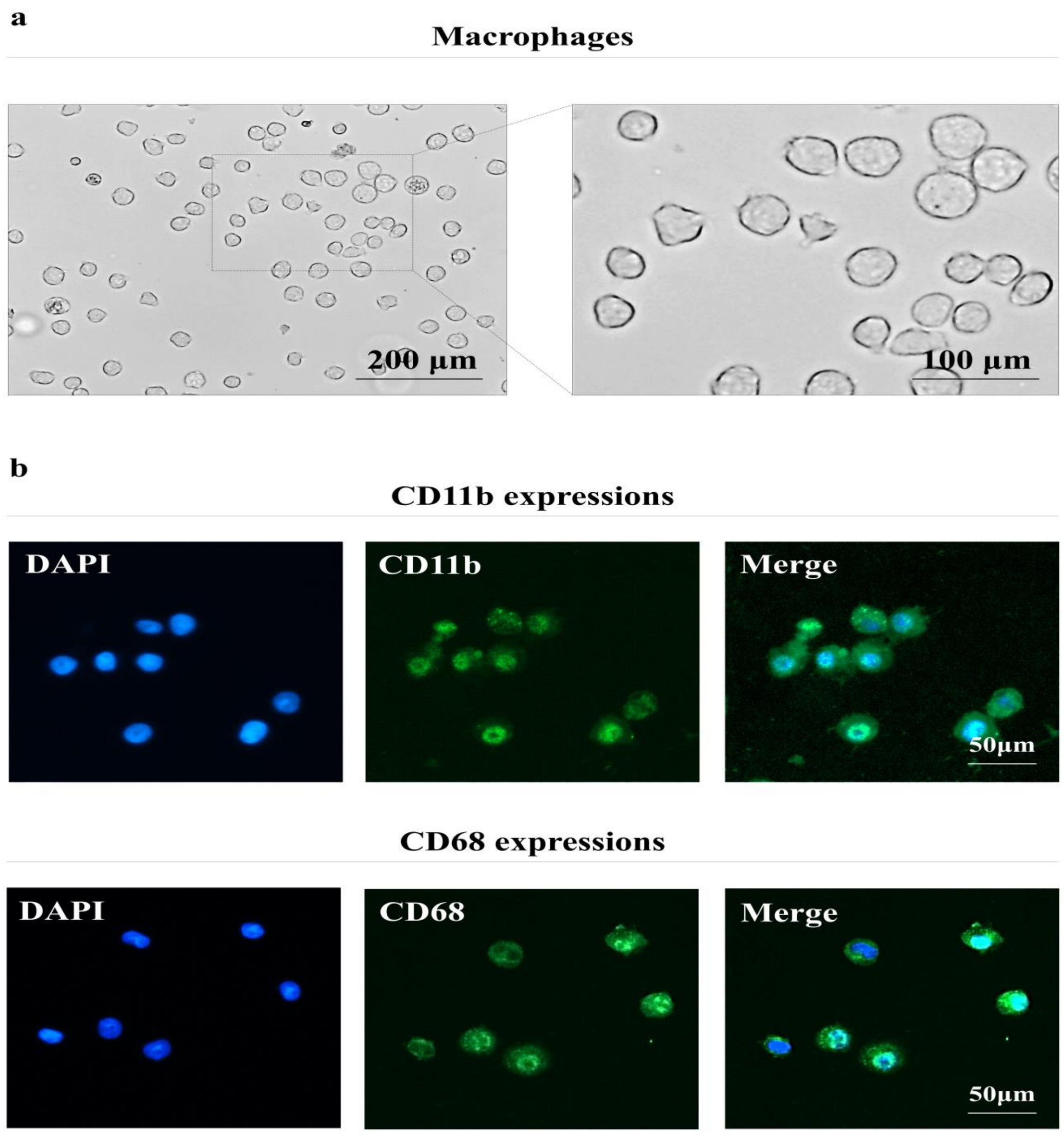

Supplementary figure 5. Characterization of human macrophages. Round-shaped human macrophages were observed by phase-contrast microscopy **(a)**. The expression patterns of putative markers CD11b and CD68 in macrophage were evaluated by immunofluorescent staining **(b)**. DAPI staining was used to label the nuclei within each field.

Supplementary figure 6

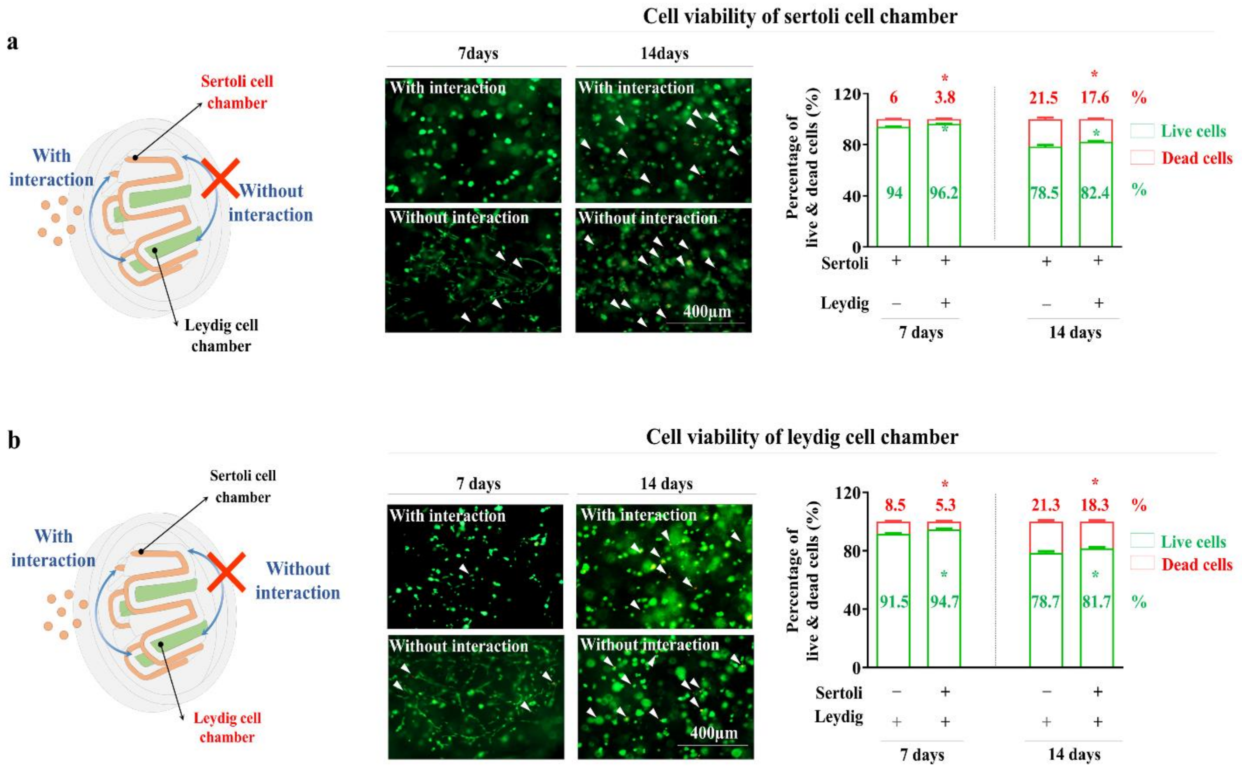

Supplementary figure 6. Effects of bidirectional endocrine crosstalk on the viability of loaded cells within two chambers. Both Sertoli and the Leydig cell chambers were incubated in standard culture medium for 7 days and 14 days with or without cross communications, and then the assay solution was added to each sample. It was confirmed that bidirectional cross communication between these chambers had no significant effect on the viability of cells loaded in the Sertoli **(a)** and Leydig cell **(b)** chambers. Significant differences are presented. $*p < 0.05$, $**p < 0.005$, and $***p < 0.001$ (two-sample t-test).

Supplementary figure 7

a

Positive correlation between apoptosis and cytotoxicity signaling pathways in Sertoli cells

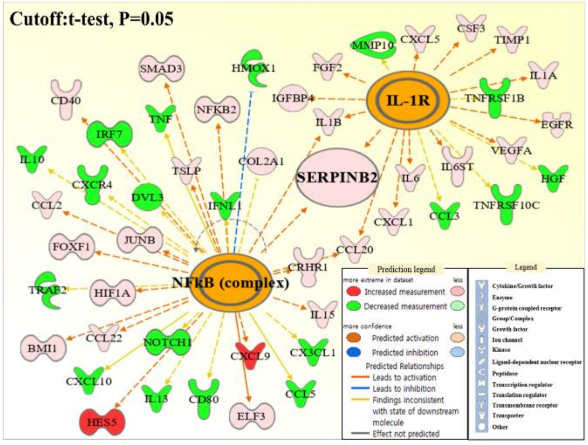

Signaling pathways	Predicted activation group	# of Target molecules in dataset	Predicted activation state	Activation z-score	P-value of overlap
**IL-1R**	Dioxin treated cells (vs Non-dioxin treated cells)	28	Activated	3.480	1.03E-04
**NFkB**	Dioxin treated cells (vs Non-dioxin treated cells)	134	Activated	2.492	1.65E-06

b

Positive correlation between apoptosis and cytotoxicity signaling pathways in Leydig cells

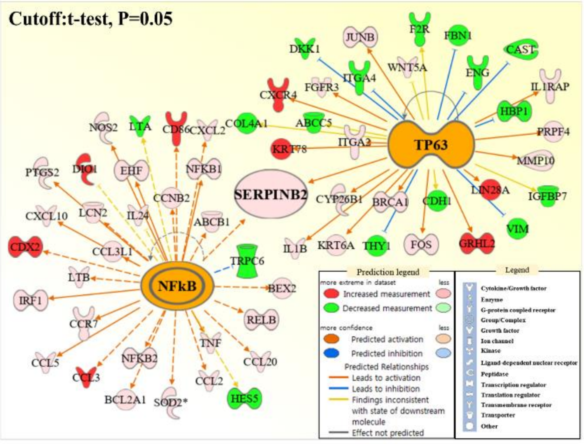

Signaling Pathways	Predicted activation group	# of Target molecules in dataset	Predicted activation state	Activation z-score	P-value of overlap
**NFkB**	Dioxin treated cells (vs Non-dioxin treated cells)	133	Activated	5.369	4.21E-06
**TP63**	Dioxin treated cells (vs Non-dioxin treated cells)	143	Activated	3.783	2.03E-05

Supplementary figure 7. Various SERPINB2-associated signaling networks were analyzed using ingenuity pathway analysis (IPA). Based on RNA-sequencing data for the toxicant (dioxin) treated and the untreated Sertoli or Leydig cells, the activation status (intermediate, inactivate, or activate) of various SERPINB2-associated genes and signaling molecules were analyzed using the IPA platform. The differentially activated status of SERPINB2-associated signaling pathways (NF-κB and IL-1R associated signaling molecules/transcription factors) between the toxicant-treated and untreated Sertoli cells were analyzed using IPA platform **(a)**. The differentially activated status of SERPINB2-associated signaling pathways (NF-κB and TP63 associated signaling molecules/transcription factors) between the toxicant treated and the untreated Leydig cells were analyzed using the IPA platform **(b)**.

Supplementary figure 8

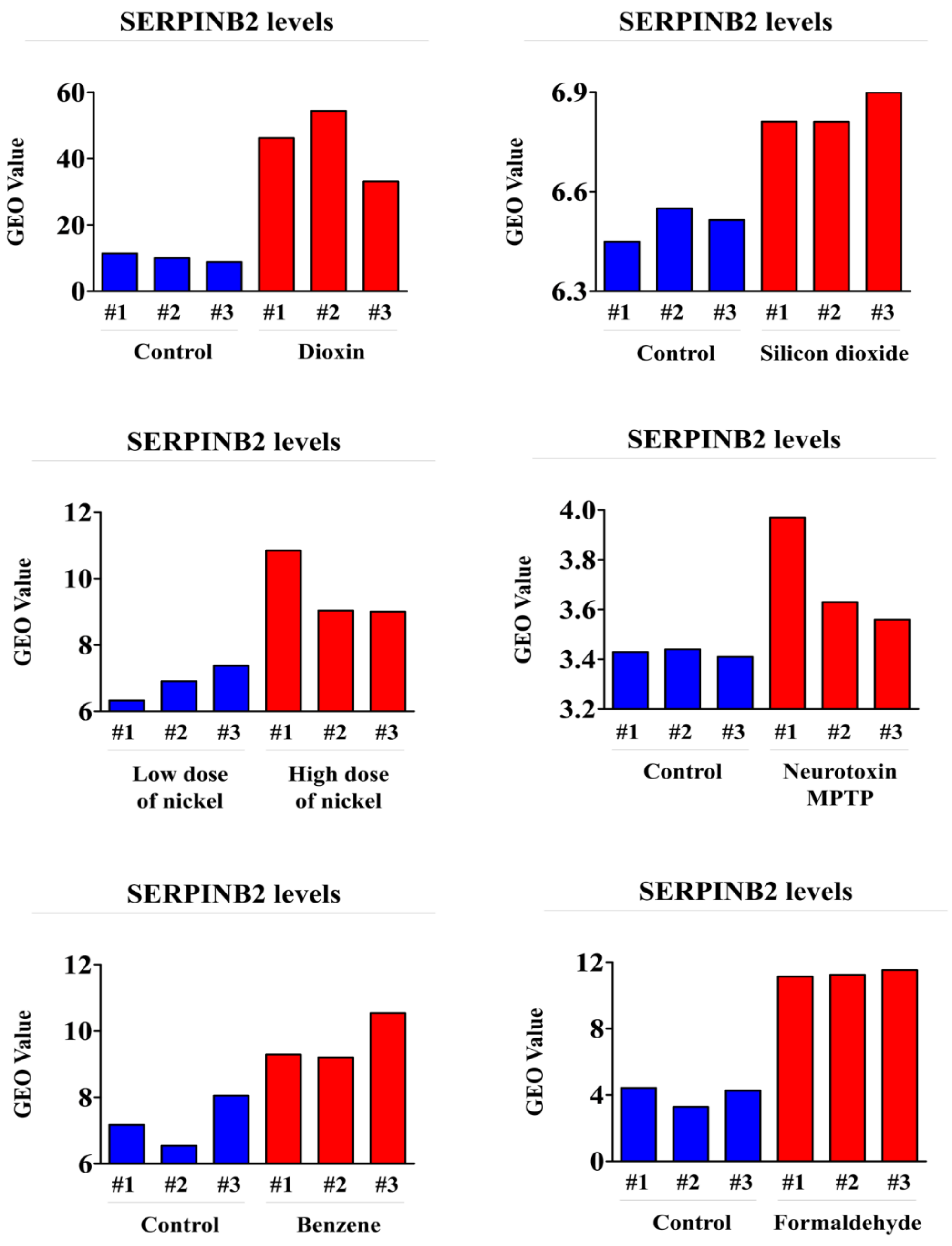

Supplementary figure 8. SERPINB2 levels are increased markedly in response to various toxic exposures. The information on Gene Expression Omnibus (GEO) public data repository was analyzed to evaluate the positive correlations between enhanced SERPINB2 levels and various toxic substance exposures.

Supplementary figure 9

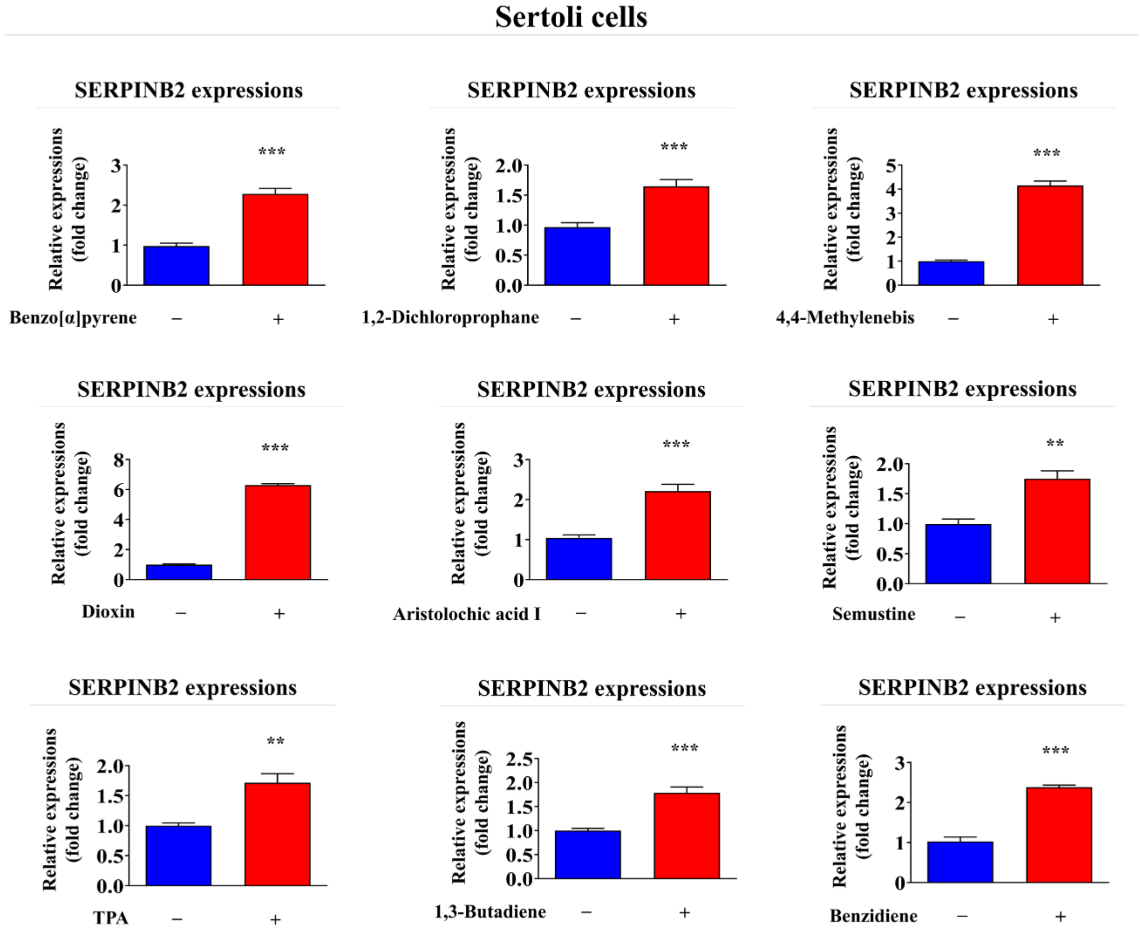

Supplementary Figure 9. Various toxic exposures significantly increase SERPINB2 expression levels in human Sertoli cells. Expression levels of SERPINB2 following exposures to various types of toxic materials, such as benzo[a]pyrene (2 μM), 1,2-dichloropropane (100 mM), 4,4'-methylenebis (5 μM), dioxin (5 ng/ml), aristolochic acid I (10 μM), semustine (0.5 mM), TPA (5 nM), 1,3-butadiene (10 mM), and benzidine (10 μM) for 72 h in Sertoli cells were determined by performing real-time PCR. *PPIA* was used as a housekeeping gene for real-time PCR. Significant differences are presented. *, $p < 0.05$; **, $p < 0.005$; and ***, $p < 0.001$ (two-sample t-test).

Supplementary figure 10

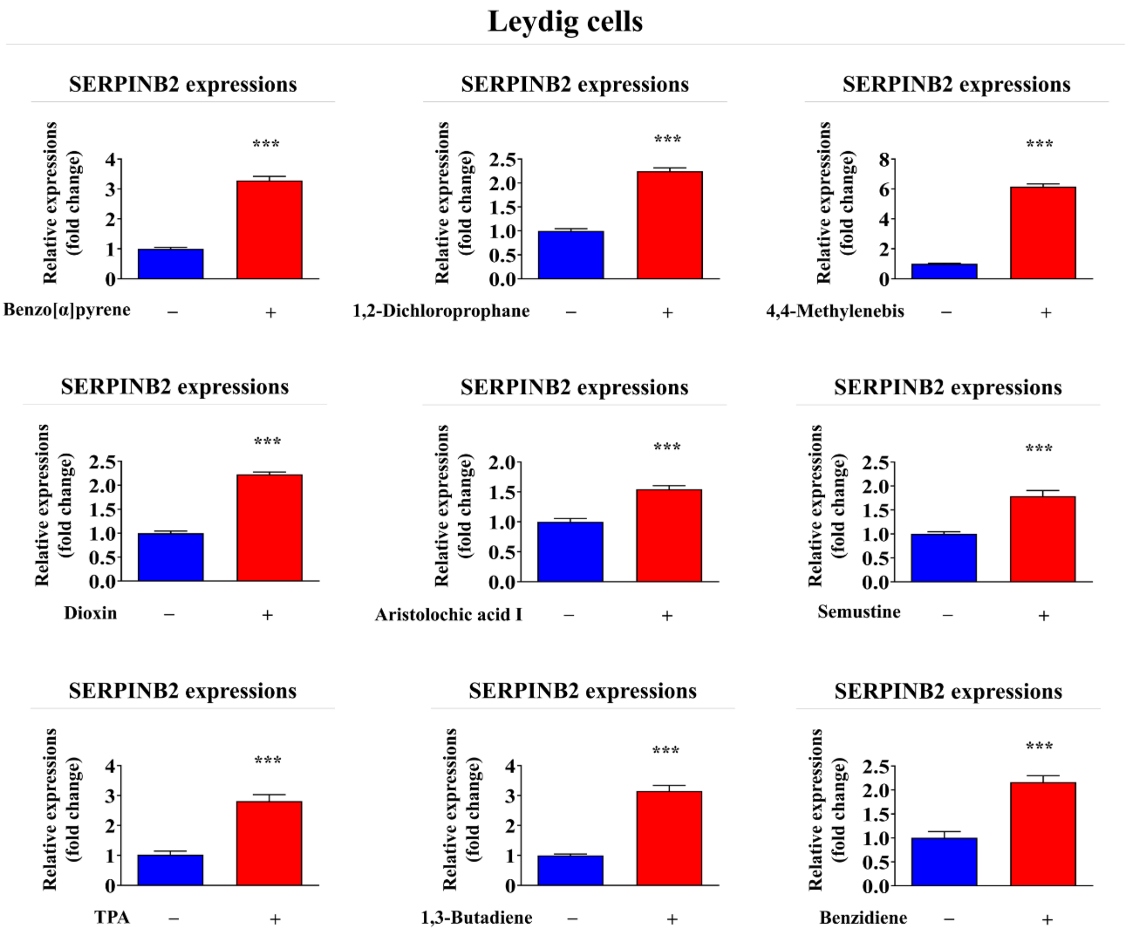

Supplementary Figure 10. Various toxic exposures significantly increase SERPINB2 expression levels in human Leydig cells. Expression levels of SERPINB2 following exposures to various types of toxic materials, such as benzo[a]pyrene (2 µM), 1,2-dichloropropane (100 mM), 4,4’-methylenebis (5 µM), dioxin (5 ng/ml), aristolochic acid I (10 µM), semustine (0.5 mM), TPA (5 nM), 1,3-butadiene (10 mM), and benzidine (10 µM) for 72 h in Leydig cells were determined by performing real-time PCR. *PPIA* was used as a housekeeping gene for real-time PCR. Significant differences are presented. *, $p < 0.05$; **, $p < 0.005$; and ***, $p < 0.001$ (two-sample t-test).

Supplementary figure 11

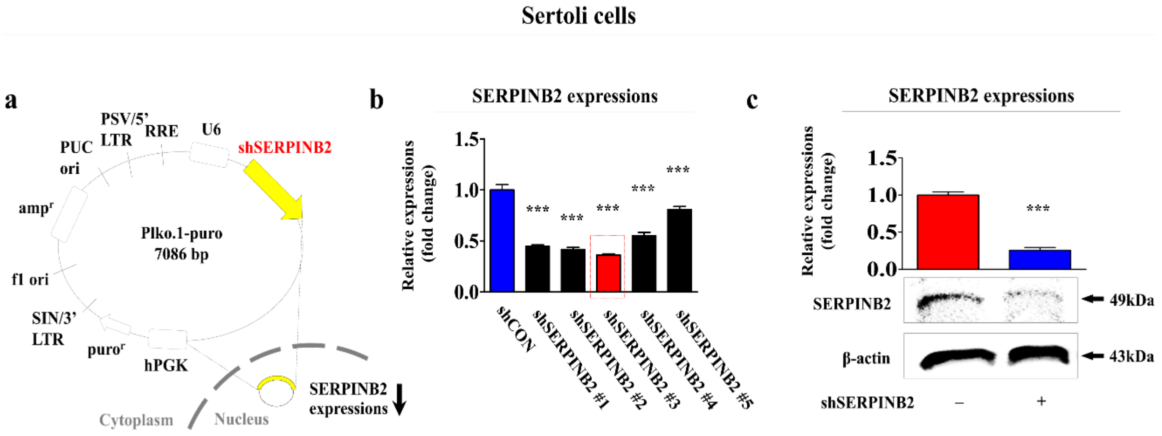

Supplementary Figure 11. Transfection efficacy of SERPINB2 knockdown using specific shRNAs in human Sertoli cells. Sertoli cells were properly transfected with various shRNAs constructs (#1, #2, #3, #5, or #4), specifically targeting SERPINB2 **(a)**. shRNA constructs #3 (hereafter referred to as SERPINB2 shRNA) depleted SERPINB2 expression most effectively at both the mRNA **(b)** and protein **(c)** levels. β-actin was used as an internal protein control. *PPIA* was used as a housekeeping gene for real-time PCR. All experiments were performed in triplicate. Data are presented as means ± standard deviations. *, $p < 0.05$; **, $p < 0.005$; and ***, $p < 0.001$ (two-sample t-test).

Supplementary figure 12

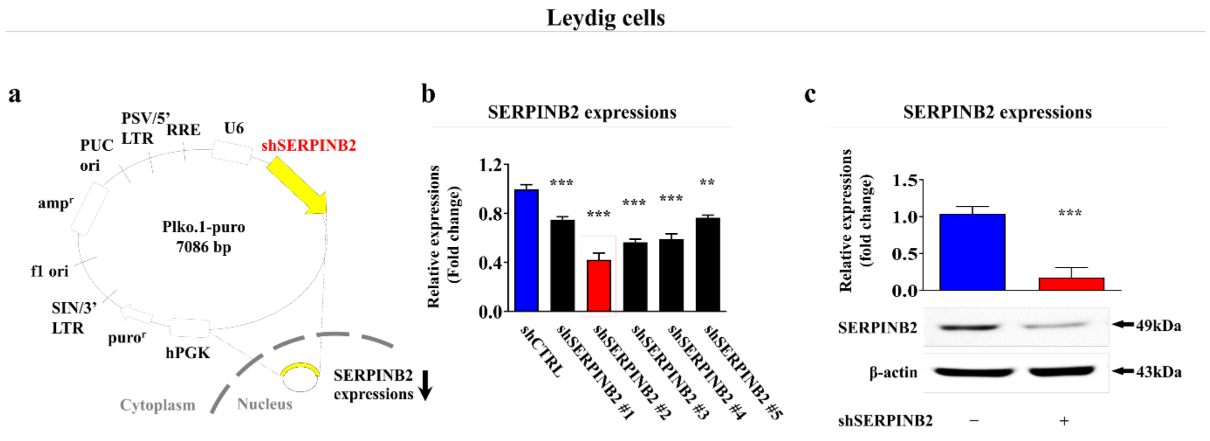

Supplementary Figure 12. Transfection efficacy of SERPINB2 knockdown using specific shRNAs in human Leydig cells. Leydig cells were properly transfected with various shRNAs constructs (#1, #2, #3, #5, or #4), specifically targeting SERPINB2 **(a)**. shRNA constructs #2 (hereafter referred to as SERPINB2 shRNA) most effectively depleted SERPINB2 expression at both mRNA **(b)** and protein **(c)** levels. β-actin was used as an internal protein control. *PPIA* was used as a housekeeping gene for real-time PCR. All experiments were performed in triplicate. Data are presented as means ± standard deviations. *, $p < 0.05$; **, $p < 0.005$; and ***, $p < 0.001$ (two-sample t-test).

Supplementary figure 13

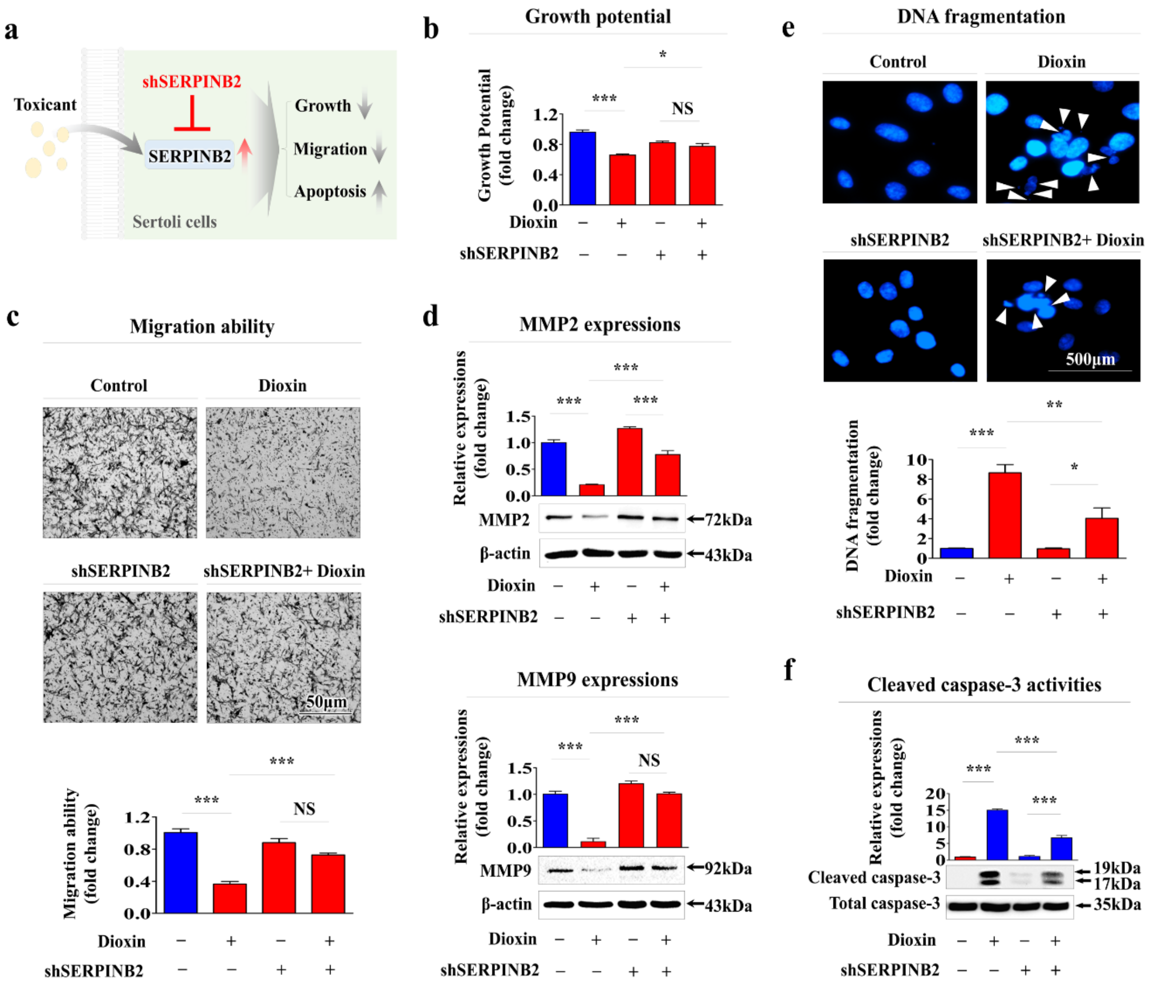

Supplementary Figure 13. Verifying the reliability of the identified biomarker (SERPINB2) for male reproductive toxicity in human Sertoli cells. Schematic diagram describing the regulatory functions of SERPINB2 that mediates toxicant-induced harmful effects in Sertoli cells **(a)**. Human Sertoli cells were transfected with a specific SERPINB2 shRNA and treated or without a well-known toxicant dioxin (5 ng/ml) for 72 h. The harmful effects on cell proliferation were analyzed using an MTT assay **(b)**. SERPINB2 depletion significantly abolished toxicant-induced harmful effects on the migratory capacity of Sertoli cells, as determined by transwell migration/invasion assay **(c)** and western blotting using antibodies against MMP-2 and MMP-9 **(d)**. Sertoli cells were transfected with a specific SERPINB2 shRNA and treated with or without a 5 ng/ml dioxin. Subsequent changes in apoptotic DNA degradation and proapoptotic caspase-3 activities were measured by nuclear staining **(e)** and western

blotting **(f)**, respectively. β-actin was used as an internal control. All experiments were performed in triplicate. Data are presented as means ± standard deviations. *, $p < 0.05$; **, $p < 0.005$; and ***, $p < 0.001$ (two-sample t-test).

Supplementary figure 14

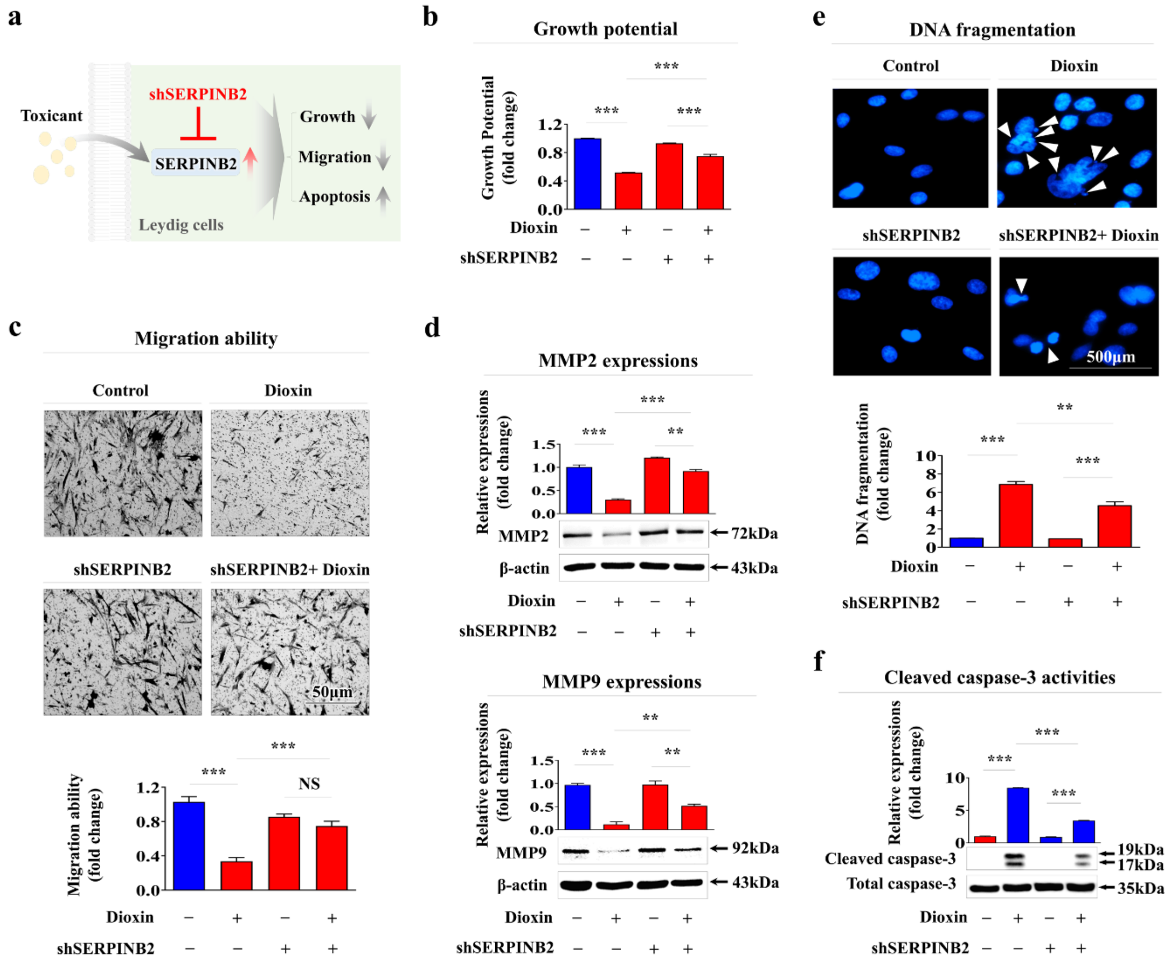

Supplementary Figure 14. Verifying the reliability of the identified biomarker (SERPINB2) for male reproductive toxicity in human Leydig cells. Schematic diagram of the regulatory functions of SERPINB2 that mediates toxicant-induced harmful effects in Leydig cells **(a)**. Human Leydig cells were transfected with a specific SERPINB2 shRNA and treated or without a well-known toxicant dioxin (5 ng/ml) for 72 h. Its harmful effects on cell proliferation were analyzed using an MTT assay **(b)**. SERPINB2 depletion significantly abolished toxicant-induced harmful effects on the migratory capacity of Leydig cells as determined by transwell migration/invasion assay **(c)** and western blotting using antibodies against MMP-2 and MMP-9 **(d)**. Leydig cells were transfected with a specific SERPINB2 shRNA and treated with or without a 5 ng/ml dioxin. Subsequent changes in apoptotic DNA degradation and proapoptotic caspase-3 activities were measured by nuclear staining **(e)** and western blotting **(f)**, respectively. β-actin was used as an internal control. All experiments were performed in

triplicate. Data are presented as means ± standard deviations. *, $p < 0.05$; **, $p < 0.005$; and ***, $p < 0.001$ (two-sample t-test).

Supplementary figure 15

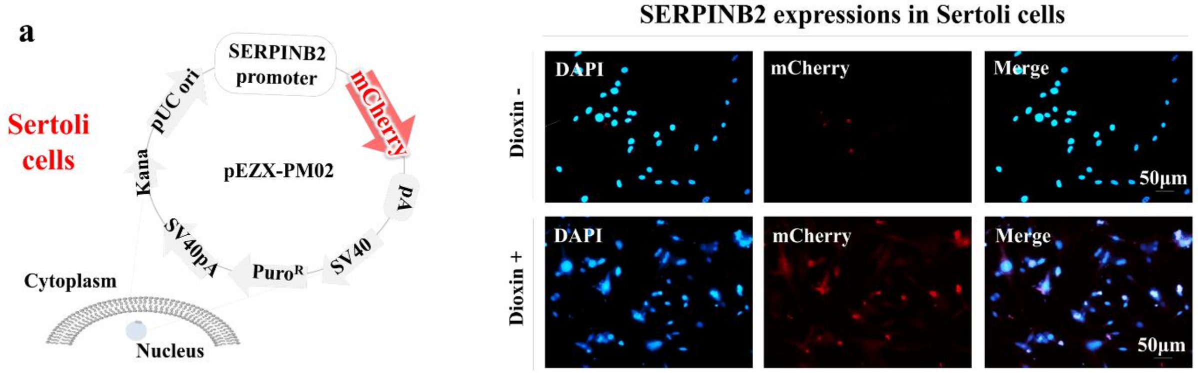

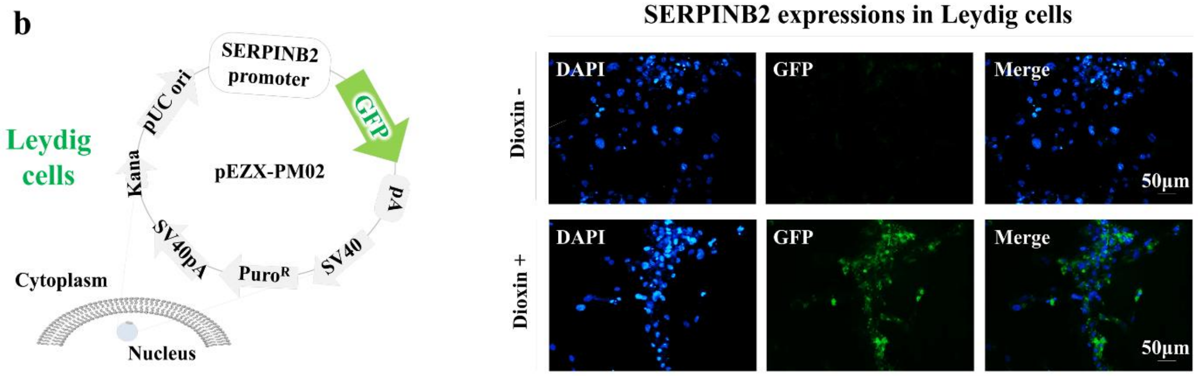

Supplementary Figure 15. Establishment of a stable SERPINB2-conjugated fluorescent reporter system for both human Sertoli and Leydig cells. Sertoli and Leydig cells were transfected stably with SERPINB2 conjugated-mCherry (red color) or GFP (green color) reporter vector, respectively. Immunostaining results showed that toxicant exposure (5 ng/ml dioxin) increased SERPINB2 activity significantly, which was subsequently converted to a red or green fluorescence signal in both Sertoli **(a)** and Leydig **(b)** cells. Significant differences are presented. *, $p < 0.05$; **, $p < 0.005$; and ***, $p < 0.001$ (two-sample t-test).

Supplementary materials and methods

Immortalization and establishment of various human testicular cellular components

In addition, normal human cells have limited growth potential *in vitro*. Therefore, the immortalized human Sertoli cells and Leydig cells with minimal alternation of their original characteristics were established by the stable transfection of SV40 large T antigen. Immortalized cells were obtained from a single clone that is more homogenous than the heterogeneous primary cultured cells with various morphologies. The culture medium was changed every 2–3 days depending on the cell confluency. Normal human umbilical vein endothelial cells (HUVECs) were obtained from ATCC (PCS-100-010) and then expanded in an EBM-2 culture medium supplemented with an EGM-2 single quote kit (Lonza). Normal human macrophages were obtained from ATCC (CRL-9855) and expanded in 10% FBS and 1% Pen/Strep-containing RPMI 1640.

Live and Dead assay for analyzing long-term cell survival rates

The long-term cell survival rates of the loaded cells within the fabricated testis-on-a-chip platform were assessed by performing a Live and Dead assay (Invitrogen and Cat. No: L3224) at one day, seven days, 14 days, 21 days, and 28 days after cell embedding according to the manufacturer's protocols. Each chip compartment was washed three times with DMEM medium without serum. A 1 ml assay solution containing 2 mM EthD-1 and 4 mM calcein AM was added to each chamber. After 30 min incubation at room temperature, each compartment was observed using an EVOS FL Cell Imaging System (Thermo Fisher Scientific, Waltham, USA).

Analysis of data from the Gene Expression Omnibus (GEO) database repository

GEO (https://www.ncbi.nlm.nih.gov/geo/) is an international public database repository of high-throughput gene expression analysis produced by chip sequencing, DNA microarrays, and RNA sequencing [24, 25]. GEO datasets provide experimental results in four sections: experimental designs, raw data, groups, and platform. The experimental results within each dataset were classified further

according to various categories, such as disease states, physiologic conditions, and treatment conditions. These classified functional data are shown as a "GEO profile", which includes functional annotation, a chart depicting value, and rank measurements for that gene across all analyzed samples [26]. The expression profiles of SERPINB2 in response to exposures to various toxic materials were analyzed according to previously established procedures [26].

Evaluation of long-term metabolic activities of loaded cells

Long-term metabolic activities of loaded cells within the testis-on-a-chip were analyzed by performing a CCK-8 assay (Abbkine and Cat. No: KTC011001) according to the manufacturer protocols. Each compartment was incubated with 100 μl/ml of a CCK-8 solution in serum-free DMEM to perform the CCK-8 assay. The cell-loaded chip was then incubated in the 5% CO_2 incubator for 4 h at 37°C. The absorbance at 450 nm was used to determine the metabolic activities of the loaded cells using the microplate reader Soft max pro 5 (Molecular device, San Jose, USA).

Ingenuity Pathway Analysis

The SERPINB2-associated signaling networks were analyzed using Ingenuity Pathway Analysis (IPA) version 2.0 software (Ingenuity Systems, Redwood City, CA). Differentially activated signaling pathways or genes (t-test, $P < 0.005$) between apoptotic cells and normal cells were analyzed using the SERPINB2- associated genes. The significance of each signaling pathway or gene was analyzed by Fisher's exact test (P value), which was used to identify the differentially activated signaling pathways or genes. The activation score (Z-score) was used to describe the status of predicted the signaling pathways or genes by comparing the observed differential regulation of the gene ("up" or "down") in the microarray data relative to the literature-derived regulation direction, which could be either activating or inhibiting.
